# Digital Augmentation of Glucagon-Like Peptide-1 for Obesity Management: Retrospective Study

**DOI:** 10.2196/100728

**Published:** 2026-11-03

**Authors:** Belinda Davey, Omar Dabash, Rahul Khanna, Portia Y Cornell, Kit Huckvale

**Affiliations:** 1 Centre for Digital Transformation of Health, Faculty of Medicine, Nursing and Health Sciences, The University of Melbourne, Level 8, Melbourne Connect, 700 Swanston St, Parkville, Victoria, 3010, Australia, 61 0405958231

**Keywords:** digital health, weight loss, GLP-1 RA medication, tirzepatide, semaglutide, self-monitoring, behavioral engagement, Glucagon-like peptide-1 receptor agonist

## Abstract

**Background:**

Glucagon-like peptide-1 receptor agonists (GLP-1 RAs), including semaglutide and tirzepatide, are highly effective for weight management; however, long-term outcomes depend on sustained behavioral engagement. Digital health tools may enhance these behavioral components, but evidence regarding their real-world benefit alongside pharmacotherapy remains limited.

**Objective:**

This study aims to evaluate whether augmenting a direct-to-consumer GLP-1 RA program with a behavioral support smartphone app improves weight-loss outcomes compared with medication alone, and to identify which app features are associated with weight loss over time.

**Methods:**

A retrospective observational cohort study was conducted using data from 6113 adults (older than 18 years) in Australia who self-enrolled into the Mosh direct-to-consumer weight-loss program between July 1, 2024, and August 13, 2025, with their weight data recorded at baseline and at least 1 other timepoint. Participants received semaglutide or tirzepatide, with optional access to a smartphone app and clinician support. Logistic regression models examined achievement of ≥5%, ≥10%, and ≥15% weight-loss thresholds at 60, 120, and 180 days. Linear mixed-effects models interacting with app feature use, with linear and quadratic time terms, assessed the association of different features with percentage weight loss trajectories over time. Both models adjusted for medication type, gender, ethnicity, age, and baseline BMI.

**Results:**

Participants using the program plus medication had higher odds of achieving clinically significant weight loss than those using medication only. Adjusted odds ratio point estimates for day 60, 120, and 180 (±10) days indicated they were 1.38 (95% CI 1.09-1.75) times to 2.22 (95% CI 1.40-3.45) times more likely to achieve ≥5% weight loss across timepoints, 1.67 (95% CI 1.33-2.10) times to 1.97 (95% CI 1.35-2.90) times more likely to achieve ≥10% weight loss by day 120 and 180, and 1.99 (95% CI 1.26-3.29) times more likely to achieve ≥15% weight loss by 180 days. Mixed-effects modeling demonstrated that progress logging was associated with more rapid weight loss over time (β=.26 percentage-point additional weight lost per 30 days, SE 0.06; *t*_4336_=4.46; 95% CI 0.14-0.38; *P*<.001). No other app features were consistently associated with changes in weight-loss trajectories.

**Conclusions:**

Augmenting GLP-1 RA treatment with a behavioral smartphone app was associated with a higher likelihood of achieving clinically significant weight loss, although differences in overall weight-loss trajectories were modest. Progress logging emerged as the primary behavioral predictor of improved outcomes. While not causal effects, these findings are consistent with the positive value of integrating digital tools with pharmacotherapy for weight loss.

## Introduction

Obesity is a complex and chronic disease increasing the risk of numerous health conditions, such as type 2 diabetes and cardiovascular disease [1,2]. Achieving and maintaining weight loss can be challenging, leading to a need for effective pharmacological interventions. Glucagon-like peptide-1 receptor agonists (GLP-1 RAs), initially developed for type 2 diabetes, have emerged as a significant therapeutic class for managing obesity due to their profound effects on weight reduction [3]. Clinical studies have consistently shown GLP-1 RAs to be effective in promoting significant weight loss in individuals with obesity, both with and those without type 2 diabetes [2,4]. Evaluations of direct-to-consumer models of care have also shown promise [5]. However, there are considerable challenges in patient adherence and persistence to these treatments [6]. These challenges often stem from side effects, accessibility issues, and a lack of integrated lifestyle support, which can undermine the long-term efficacy of these pharmaceutical interventions [7].

Digital health tools such as smartphone apps are widely used, for example, for mental health issues and health behavior change, as they allow contextual, accessible, scalable, and personalized support (eg [8,9]). Such tools can overcome traditional barriers to care, enhancing patient engagement and offering continuous support beyond conventional clinical settings [1]. They may also be useful for augmenting weight-loss medications to help achieve sustained behavior change. However, the clinical benefit of such apps is unclear and requires careful examination. Nonetheless, there is some limited evidence that digital health apps combined with pharmacological treatments may optimize patient outcomes by fostering greater adherence, providing personalized feedback, and facilitating sustained behavioral changes necessary for effective obesity management [6].

Several targets have been explored in the literature. First, self-monitoring, including logging of weight, dietary intake, and physical activity, has been identified as a critical behavior change technique (BCT) [10]. Days with food logging and regular weight entries show a robust dose-response relationship, whereby more frequent self-monitoring predicts greater weight loss and better maintenance [11-13].

Second, there is evidence for targeting goal setting, ongoing feedback, and personalized targets. Digital programs that combine self-monitoring with goal review, progress feedback, and adaptive coaching produce greater weight loss than those offering static educational content [14-17]. Smartphone-based interventions that deliver real-time data on calorie balance, physical activity, or weight trajectory show enhanced behavior regulation and adherence [18].

Third, research consistently shows that reminders and digital prompts, such as push notifications, SMS nudges, or in-app cues, may help to sustain engagement and prevent early disengagement from digital programs [19,20]. Engagement metrics such as days logged in to the app, meals logged, or sessions completed are among the strongest predictors of clinically significant weight loss [21-23]. In GLP-1 programs augmented by digital applications, higher digital engagement correlates with greater weight loss (eg, differences of 3‐3.5 percentage points at 3‐5 months between high- and low-engagement groups [24,25]).

Some evidence suggests that human support, whether from dietitians, nurses, health coaches, or peer communities, may enhance digital program effectiveness [17,26]. Programs that integrate human support have shown higher adherence, greater satisfaction, and larger reductions in weight than programs that are digital only. In digital-pharmacologic hybrid models (eg, GLP-1 RA programs such as Voy, Second Nature, and Embla), human interaction is explicitly associated with greater weight loss over and above pharmacotherapy alone [25,27,28].

Finally, some studies highlight the role of healthy meal exposure, recipe tools, and food planning features in supporting dietary adherence. Apps that integrate recipe libraries, personalized suggestions, or meal planning show improved user satisfaction and better long-term retention, facilitating consistent calorie reduction [29]. Although these features tend to play a supportive rather than primary role, they contribute to sustained dietary adherence.

In summary, emerging evidence suggests direct-to-consumer obesity management programs are effective, and that digital augmentation may enhance effectiveness. The primary aim of this study was to evaluate whether augmenting a direct-to-consumer GLP-1 RA program with a behavioral support smartphone app (Mosh) is associated with improved weight loss outcomes compared with medication alone. A secondary aim was to examine which app features are most strongly associated with weight loss.

## Methods

### Study Design

We conducted a retrospective, noninterventional cohort study using anonymized, self-reported clinical outcome data from Australian program participants (n=8148) self-enrolled into the Mosh direct-to-consumer weight-loss program between July 1, 2024, and August 13, 2025, who had enrolled at least 180 days prior to the date of data extraction (as the program runs for 180 days). Participants were categorized into the medication-only group (n=2683) or the app+ medication group (n=5465) according to whether they voluntarily use program features (the smartphone app and nurse and dietitian consult). All participants were required to engage with mandatory, monthly clinical check-ins with clinicians (nurses or doctors) to ensure patients are achieving ongoing weight loss and that the medication remains clinically safe. Unlike most other published studies [25,27,30], there are no weight or comorbidity restrictions for participants enrolling in this study.

### Measures

Baseline weight, height, BMI, ethnicity, gender, initial GLP-1 drug and dose, and program start date (that is, when the program participant was first approved as suitable for the program) were recorded at day 0. Subsequently, participants were invited to provide their current weight every 30 days (with a 10-day leeway; eg, 20 to 40 days), and were required to provide their current weight upon each GLP-1 RA script renewal, which was typically at 30 and 60 days, and then every 30 to 60 days thereafter, depending on clinical need. Participants used their own scales to weigh themselves and self-reported their weight. Not all participants provided data every 30 days. Participant BMI and weight change were calculated from current and previous weight. The final GLP-1 RA drug and dose were also recorded, as well as subscription cancellation date, if applicable. The variables “initial dose” and “final dose” were created by categorizing roughly equivalent doses across medication types into dose levels 1, 2, 3, 4, and 5, which were semaglutide 0.25 mg and tirzepatide 2.5 mg, semaglutide 0.5 mg and tirzepatide 5 mg, semaglutide 1 mg and tirzepatide 5 mg, semaglutide 1 mg and tirzepatide 7.5‐10 mg, semaglutide 1.7 mg and tirzepatide 12.5 mg, and, finally, semaglutide 2.4 mg and tirzepatide 15 mg, respectively.

### Procedure

The 180-day program prescribed GLP-1 RA weight-loss medications to all enrolled participants, who could choose from either tirzepatide or semaglutide and could switch medications if clinically appropriate. All participants started on either 0.25 mg-0.5 mg of injectable semaglutide or 2.5 mg of injectable tirzepatide, and they could increase this dose throughout the program as needed and tolerated.

All participants had the option of using Mosh program features beyond medication, which entailed the use of the Mosh smartphone app and an optional online check-in with a nurse and a dietitian (to determine calorie targets, discuss diet habits, and develop a personalized diet plan). Check-ins could be synchronous (ie, a live consult) or asynchronous, and may include, for example, lifestyle advice, discussion regarding the patient’s diet, and/or a personalized behavioral plan to help the patient achieve their weight loss goals. App features comprised setting target calories; setting dietary and weight-loss goals; planning, logging and scanning meals; logging progress (at additional timepoints outside of clinical appointments); “favoriting” a recipe; and viewing recipes.

### Defining Intervention Groups

All participants who used at least one app feature at least once (which could include a nurse or dietitian check-in) were allocated to the app+medication group; all other participants (who used either tirzepatide or semaglutide, but did not engage with the Mosh program), were categorized into the medication only group. No information was available regarding the regularity of feature use (ie, the variables were binary, indicating if a feature had been used by a participant, with no information about regularity).

### Analytic Plan

#### Data Cleaning

Participants who provided only baseline weight data or implausible height values (<100 cm) were removed from analyses, as the latter makes BMI data (which was included as a covariate) unreliable. Implausible consecutive 30-day changes in BMI (>5) or percentage weight loss (±15%) were set to missing, as were implausible percentage weight loss values (>20% within 90 days, and progressively higher thresholds of >25%, >30%, and >35% at 120, 150, and 180 days, respectively [31-33]).

#### Attrition and Cancellation Analyses

To assess potential selection bias arising from missing follow-up data, participants with baseline-only data were compared with those who provided at least one postbaseline weight measurement. Baseline characteristics were summarized by follow-up data status, and between-group differences were quantified using standardized mean differences with 95% CIs, as trivial differences can yield significant *P* values in a large sample. Absolute standardized mean differences below 0.10 were interpreted as indicating negligible imbalance, consistent with commonly used covariate-balance guidance [34], while values from 0.10 to below 0.20 were considered small and values of 0.20 or greater were considered potentially meaningful, informed by Cohen [35] conventional benchmark for a small standardized effect. A similar analysis was conducted to compare participants who canceled their subscription before 180 days with those who remained enrolled.

These analyses are intended to identify whether attrition or cancellation are systematically associated with baseline demographic, anthropometric, treatment, or program characteristics. Because program participation is defined by observed use of app features, differences in program classification are interpreted cautiously, as participants without follow-up data or who canceled early also had fewer opportunities to demonstrate app engagement.

#### Clinically Significant Weight Loss

Clinically significant weight loss was examined via a series of logistic regressions, examining 5%, 10%, and 15% clinically significant weight loss thresholds at 60, 120, and 180 days by intervention group (percentage weight loss was calculated by comparing to baseline weight for each timepoint, rather than to the previous timepoint), statistically adjusting for age, gender, ethnicity, baseline BMI, and medication type. These weight-loss thresholds were selected based on established clinical guidelines and trial conventions [25,27,28,31,36,37]. A ≥5% reduction represents the minimum threshold associated with meaningful improvements in cardiometabolic risk factors, including glycemic control, blood pressure, and lipid profiles [32,38,39], while ≥10% reflects more substantial disease modification and greater improvements across metabolic, cardiovascular, and hepatic outcomes [38]. The ≥15% threshold has emerged in recent pharmacotherapy trials as an indicator of high treatment response, particularly in studies of semaglutide and tirzepatide and is often associated with remission of obesity-related comorbidities, including type 2 diabetes [31,36,37]. Together, these thresholds capture a graded spectrum of clinically meaningful outcomes and are widely used in obesity research and clinical practice, in the absence of evidence for interaction between program participation with medication type or demographics (age, gender, and ethnicity) when predicting percentage weight loss at day 60, 120, or 180 in this study.

#### Model Diagnostics

Model diagnostics were conducted for each logistic regression model. Multicollinearity was assessed using variance inflation factors (VIFs). Influential observations were assessed using Cook’s distance of 1 as a cutoff for influential outliers.

#### Inverse Probability-of-Censoring Weighted Sensitivity Analyses

To assess the sensitivity of the adjusted logistic regression results to differential loss to follow-up, we conducted inverse probability-of-censoring weighted (IPCW) analyses. For each follow-up timepoint, a logistic regression model was used to estimate each participant’s probability of having an observed weight-loss outcome from program group, age, gender, ethnicity, baseline BMI, and initial medication type. Participants who did provide follow-up data but had characteristics associated with a lower likelihood of being observed were given more influence in the analysis, so that they could represent similar participants whose outcomes were missing. Separate weights were calculated for days 60, 120, and 180. We also checked whether any participants received unusually large weights and repeated the analyses after limiting the most extreme weights. This provided an additional check on whether the findings were sensitive to loss to follow-up.

#### Quantitative Bias Analysis for Unmeasured Confounding

Because participants self-selected into the app+medication group, E-value sensitivity analyses were conducted to assess the potential influence of unmeasured confounding. For each adjusted odds ratio (OR) greater than 1, the E-value was calculated as follows:


$$\text{E}\text{-}\text{value}\text{=}\text{OR}\text{+}\sqrt{\text{OR}\text{(}\text{OR}\text{- 1)}}$$

where OR is the adjusted OR. The E-value represents the minimum strength of association, on the risk-ratio scale, that an unmeasured confounder would need to have with both program participation and the weight-loss outcome, after accounting for the measured covariates, to fully explain the observed association. E-values were calculated for both the point estimate and the confidence limit value closest to the null. Larger E-values indicate that stronger unmeasured confounding would be required to explain the observed association.

#### Kaplan-Meier Survival Curves

As a complementary descriptive time-to-event analysis, Kaplan-Meier curves were used to describe time to first achievement of ≥5%, ≥10%, and ≥15% weight loss. For each threshold, the event was defined as the first recorded timepoint at which the participant met or exceeded the specified percentage weight-loss threshold. Participants who did not achieve the threshold during follow-up were censored at their last available weight measurement. Curves were stratified by intervention group to visually compare the timing and cumulative probability of achieving each weight-loss milestone.

#### Examining Which App Features Predict Weight Loss Over Time

To examine change in percentage weight loss over time in the app+medication group, percentage weight loss was modeled using linear mixed-effects models. Time was represented using study phase, coded in 30-day units from baseline, with phase 0 corresponding to baseline and subsequent phases corresponding to 30-day intervals through 180 days. Both linear and quadratic effects of phase were included to allow for nonlinear change in percentage weight loss over follow-up. App-feature indicators were mean-centered prior to model estimation to aid interpretation and reduce nonessential collinearity in interaction terms. To reduce overfitting and multicollinearity, app features were first examined in separate adjusted feature-specific models. Features showing evidence of association with baseline percentage weight loss and/or percentage weight-loss trajectory, together with clinically interpretable candidate features, were retained in the reduced multivariable model. To determine whether associations between individual app features and weight-loss trajectories varied nonlinearly, separate feature-specific mixed-effects models were fitted with and without a feature-by-quadratic-time interaction. Nested models were compared using likelihood ratio tests and changes in Akaike information criterion (AIC). Quadratic feature interactions were retained only where they improved model fit.

#### Model Diagnostics

Model diagnostics were conducted using performance::check_model(), which provides a panel of visual diagnostic checks for model assumptions, including the observed versus model-predicted distribution, residual linearity, homogeneity of residual variance, residual normality, random effect normality, and influential observations. Multicollinearity was assessed separately using VIFs from performance::check_collinearity() and Cook’s distance cutoff of 1. Inverse-probability weighted sensitivity analyses were also conducted to evaluate the impact of dropout and survivor bias on the model.

#### IPCW Sensitivity Analyses

To examine whether missing follow-up measurements or early cancellation may have affected the longitudinal results, we conducted an IPCW analysis. For each recorded observation, we estimated how likely the participant was to remain in follow-up and to have a weight measurement available, based on time, program group, baseline BMI, age, ethnicity, and gender. Observations from participants who remained in the study despite having characteristics associated with a lower likelihood of continued follow-up or an available weight measurement were given greater influence in the analysis, so that they could represent similar participants whose later data were missing. The 2 weights were combined for each observation. We checked whether any observations received unusually large weights and limited the most extreme values at the first and 99th percentiles. The weighted mixed-effects model otherwise used the same structure as the primary model, and its results were compared with the original unweighted analysis to assess whether the findings were sensitive to dropout and missing follow-up weight data.

### Ethical Considerations

The University of Melbourne Human Research Ethics Committee (HREC) provided approval to conduct this study using data already collected by Hairmop and ensuring participant privacy and confidentiality (project ID 33938). The HREC provided a waiver for the requirement to reconsent participants, allowing the research team to use the privacy policy consent signed by program participants when subscribing to Mosh, which included consent to use their personal data for research.

## Results

### Data Cleaning

Several participants (2035/8,172, 24.9%) reported weight-loss data at day 0 only, so they could not be included in analyses. Another 10 participants who recorded heights <100 cm, producing BMI>80 kg/m^2^, were removed from the dataset. In addition, 20 weight values corresponding to 30-day BMI change >5 were set to missing and 148 BMI and weight values and 105 percentage weight-loss values associated with 30-day percentage-weight-loss values exceeding ±15%. Implausible percentage weight-loss values (>20% within 90 days, and progressively higher thresholds of >25%, >30%, and >35% at 120, 150, and 180 days, respectively) were set to missing for 71 observations [31,33,37]. Only participants with baseline weight data and weight data at at least 1 other timepoint were retained for analyses. This left 6113 of the initial 8148 participants who enrolled in the program 180 days prior to data extraction. This data cleaning resulted in sufficiently symmetric distributions for each percentage weight-loss value, and no influential outliers.

### Attrition and Cancellation Analysis

#### Overview

A dropout analysis was conducted to compare participants with baseline-only data (n=2035) against those with more than baseline data (n=6113) using standardized mean differences (Table 1). Table 1 shows that all standardized mean differences on all characteristics were negligible (<.10) or small (<0.20), other than program group. Almost two-thirds of those in the medication-only group provided only baseline data, whereas only around one-third of those in the app+medication group provided only baseline data.

**Table 1.** Comparison of participants with baseline-only data after data cleaning to those included in the analysis^a^.

Characteristics	N	Overall,	Baseline only	More than baseline	SMD^b^ (95% CI)
Baseline weight (kg), mean (SD)	8148	96.92 (18.9)	96.80 (20.28)	96.96 (18.42)	0.008 (−0.044 to 0.061)
Baseline BMI (kg/m²), mean (SD)	8148	34.03 (5.38)	34.20 (5.99)	33.97 (5.16)	−0.04 (−0.088 to 0.012)
Age (years), mean (SD)	8143	38.05 (10.39)	36.88 (10.86)	38.44 (10.2)	0.149 (0.097 to 0.202)
Gender, n (%)	8148				−0.037 (−0.083 to 0.013)
Female		5755 (70.6)	1463 (71.9)	4292 (70.2)	
Male		2393 (29.4)	572 (28.1)	1821 (29.8)	
Ethnicity, n (%)	8146				0.084 (0.036 to 0.136)
Aboriginal or Torres Strait Islander		235 (2.9)	59 (2.9)	176 (2.9)	
African		96 (1.2)	33 (1.6)	63 (1)	
Asian		420 (5.2)	120 (5.9)	300 (4.9)	
Caucasian		6279 (77.1)	1514 (74.4)	4765 (78)	
Latino or Hispanic		133 (1.6)	37 (1.8)	96 (1.6)	
Middle Eastern		181 (2.2)	62 (3)	119 (1.9)	
Not listed		626 (7.7)	163 (8)	463 (7.6)	
Pacific Islander or Maori		176 (2.2)	47 (2.3)	129 (2.1)	
Program, n (%)	8148				0.829 (0.783 to 0.876)
Medication only		2683 (32.9)	1297 (63.7)	1386 (22.7)	
App+medication		5465 (67.1)	738 (36.3)	4727 (77.3)	

a standardized mean difference–this describes the size of the difference in standardized deviation units; standardized mean difference of <0.10=negligible imbalance, 0.10‐0.20=small imbalance, and >0.20=potentially meaningful imbalance.

b SMD: standardized mean difference.

Although the possibility of attrition bias cannot be excluded, the dropout analysis provides reassurance that participants with baseline-only data were not meaningfully different from those with follow-up data on baseline weight, BMI, gender, or ethnicity. Therefore, exclusions due to baseline-only weight data are unlikely to have introduced major bias with respect to baseline anthropometric status or sex. However, because baseline-only participants appeared less engaged with the program overall, findings involving app engagement should be interpreted as applying primarily to participants who provided follow-up weight data and had observable engagement with the platform.

A similar analysis was conducted for those who actively canceled their subscription prior to being in the program for 180 days (5097/8148, 63%), and the results were very similar, though around three-quarters (2039/2697, 75.6%) in the medication-only group canceled, while only around half (3058/5451, 56.1%) in the app+medication group canceled (Section S1 in Multimedia Appendix 1 for the full analysis).

#### Participant Characteristics

Demographic characteristics were examined by intervention group (Table 2). All standardized mean differences between groups were small, other than gender (0.29). A greater proportion of those in the app+medication group were female (70.2%) compared to those in the medication-only group (59.7%).

**Table 2.** Demographic characteristics by intervention group. Continuous variables are presented as mean and SD; categorical variables are presented as n and %. SMDs^a^ are shown instead of *P* values.

Characteristic	Medication only	App+ medication	Whole sample	SMD (95% CI)
Initial weight (kg), mean (SD)	97.95 (18.93)	96.67 (18.26)	96.96 (18.42)	0.07 (0.01-0.13)
Initial BMI (kg/m^2^), mean (SD)	33.66 (4.84)	34.06 (5.25)	33.97 (5.16)	−0.08 (−0.14 to −0.02)
Height (cm), mean (SD)	170.23 (10.55)	168.21 (9.3)	168.67 (9.63)	0.2 (0.14 to 0.27)
BMI (kg/m^2^) category, n (%)				0.09 (0.04 to 0.15)
<20	1 (0.1)	0 (0)	1 (0)	
20–<25	1 (0.1)	2 (0)	3 (0)	
25–<30	242 (17.5)	812 (17.2)	1054 (17.2)	
30–<35	748 (54)	2383 (50.4)	3131 (51.2)	
≥35	394 (28.4)	1530 (32.4)	1924 (31.5)	
Gender, n (%)				0.29 (0.23 to 0.34)
Female	828 (59.7)	3464 (73.3)	4292 (70.2)	
Male	558 (40.3)	1263 (26.7)	1821 (29.8)	
Ethnicity^b^, n (%)				0.05 (0.03 to 0.11)
Aboriginal or Torres Strait I	32 (2.3)	144 (3)	176 (2.9)	
African	13 (0.9)	50 (1.1)	63 (1)	
Asian	74 (5.3)	226 (4.8)	300 (4.9)	
Caucasian	1086 (78.4)	3679 (77.9)	4765 (78)	
Latino/Hispanic	17 (1.2)	79 (1.7)	96 (1.6)	
Middle Eastern	31 (2.2)	88 (1.9)	119 (1.9)	
Not listed	105 (7.6)	360 (7.6)	465 (7.6)	
Pacific Islander or Maori	29 (2.1)	100 (2.1)	129 (2.1)	
Final drug, n (%)				0.12 (0.06 to 0.18)
Semaglutide	1008 (72.7)	3184 (67.4)	4192 (68.6)	
Tirzepatide	378 (27.3)	1543 (32.6)	1921 (31.4)	
Final dose (mg), n (%)				0.1 (0.05 to 0.16)
Semaglutide 0.25/ tirzepatide 2.5	141 (10.2)	458 (9.7)	599 (9.8)	
Semaglutide 0.5/ tirzepatide 5	354 (25.5)	1199 (25.4)	1553 (25.4)	
Semaglutide 1/tirzepatide 7.5‐10	648 (46.8)	2069 (43.8)	2717 (44.4)	
Semaglutide 1.7/tirzepatide 12.5	162 (11.7)	605 (12.8)	767 (12.5)	
Semaglutide 2.4/tirzepatide 15	81 (5.8)	396 (8.4)	477 (7.8)	

a Standardized mean differences.

b Ethnicity categories were defined by the sponsor.

The average weight and percentage weight change by program was examined stratified by medication type at each timepoint (Table 3). While these are descriptive statistics only, they show greater average weight loss for the app+medication group at each time point (Section S2 in Multimedia Appendix 1 for percentage weight loss stratified by gender).

**Table 3.** Descriptive statistics for weight and percentage weight loss by medication type and intervention group.

Medication only					App+medication		
Day and medication		n	Weight (kg), mean (SD)	Percentage weight loss, mean (SD)	n	Weight (Kg), mean (SD)	Percentage weight loss, mean (SD)
Day 0							
Semaglutide		1008	98.27 (19.04)	—^a^	3184	96.2 (17.84)	—
Tirzepatide		378	97.10 (18.63)	—	1543	97.4 (19.09)	—
Day 30							
Semaglutide		479	96.86 (19.53)	2.06 (3.24)	2202	94.2 (17.64)	2.23 (2.89)
Tirzepatide		236	95.76 (18.86)	2.42 (3.00)	1145	95.4 (19.07)	2.59 (3.17)
Day 60							
Semaglutide		559	94.24 (18.58)	4.15 (4.12)	2276	91.8 (17.64)	4.47 (3.67)
Tirzepatide		190	92.57 (20.33)	5.02 (3.97)	1038	(18.19)	5.75 (4.16)
Day 90							
Semaglutide		441	94.34 (18.51)	5.74 (4.60)	1814	90.0 (17.99)	6.58 (4.27)
Tirzepatide		147	89.31 (17.88)	7.34 (4.51)	854	90.0 (18.14)	8.18 (5.11)
Day 120							
Semaglutide		313	90.02 (17.79)	7.33 (4.89)	1429	88.2 (17.24)	8.4 (4.91)
Tirzepatide		127	87.25 (16.42)	8.81 (4.57)	735	(18.13)	9.57 (5.68)
Day 150							
Semaglutide		252	88.96 (18.82)	9.22 (5.82)	1149	(17.46)	9.91 (5.59)
Tirzepatide		97	88.73 (21.18)	9.20 (6.55)	631	86.3 (17.46)	11.64 (6.06)
Day 180							
Semaglutide		86	88.14 (16.60)	8.94 (6.22)	560	84.5 (16.05)	11.38 (6.07)
Tirzepatide		43	84.10 (17.35)	9.24 (7.24)	312	85.7 (17.39)	11.99 (6.94)

a Not applicable.

### Primary Aim: Intervention Outcomes

#### Overview

Model diagnostics for the binary logistic regressions predicting clinically significant weight-loss thresholds of 5%, 10%, and 15% at 60, 120, and 180 days indicated no evidence of problematic multicollinearity. VIFs ranged from 1.01 to 1.07 across predictors and models and all Cook’s distance values remained small (0.005-0.093) with none approaching the conventional threshold of 1.00 for high influence.

A series of binary logistic regressions found that, after statistically adjusting for age, baseline BMI, gender, ethnicity, and initial medication type (semaglutide or tirzepatide), the participants in the app+medication group were more likely than the medication-only group to achieve the 5%, clinically significant weight-loss threshold at all timepoints examined (day 60, 120, and 180), but only at day 120 and 180 for the 10% threshold and only at day 180 for the 15% threshold (Table 4). These significant adjusted OR point estimates varied from 1.38 to 2.22. The full results with all covariates are available in Section S3 in Multimedia Appendix 1 (results are also shown with inclusion of dose level in Section S3; results are very similar with or without dose level as a predictor).

**Table 4.** Results of logistic regressions comparing proportions meeting clinical weight loss thresholds for app+medication group versus medication only, adjusting for age, gender, ethnicity, initial medication type, and baseline BMI^a^.

Timepoint	Model, N	Adjusted OR^b^ (95% CI)	Z	*P* value	Medication only, n/N (%)	App+medication, n/N (%)
5% threshold						
Day 60	4063	1.40 (1.19-1.66)	3.98	<.001	295/749 (39.4)	1559/3314 (47.0)
Day 120	2604	1.38 (1.09-1.75)	2.71	.007	317/440 (72.0)	1691/2164 (78.1)
Day 180	1001	2.22 (1.40-3.45)	3.46	<.001	96/129 (74.4)	753/872 (86.4)
10% threshold						
Day 60	4063	1.05 (0.80-1.39)	0.33	.74	71/749 (9.5)	325/3314 (9.8)
Day 120	2604	1.67 (1.33-2.10)	4.42	<.001	129/440 (29.3)	887/2164 (41.0)
Day 180	1001	1.97 (1.35-2.90)	3.48	<.001	57/129 (44.2)	529/872 (60.7)
15% threshold						
Day 60	4063	0.70 (0.36-1.47)	−1.00	.32	11/749 (1.5)	34/3314 (1.0)
Day 120	2604	1.27 (0.89-1.84)	1.28	.20	39/440 (8.9)	242/2164 (11.2)
Day 180	1001	1.99 (1.26-3.29)	2.82	.005	23/129 (17.8)	262/872 (30.0)

a Odds ratios compare the app+medication group with the medication-only reference group and are adjusted for age, gender, ethnicity, baseline BMI, and initial medication type. Model n represents the complete-case analytic sample used in each adjusted regression. The group-specific frequencies and percentages are descriptive results for the same complete-case samples used in the corresponding regression models.

b OR: odds ratio.

##### IPCW Sensitivity Analyses

For the IPCW sensitivity analyses, the untruncated stabilized weights were generally well behaved. At day 60, IPCW-adjusted ORs ranged from 0.71 to 1.42; at day 120, from 0.72 to 1.42 and at day 180, from 2.03 to 2.40. The weighted estimates were substantively similar to those from the original adjusted logistic regressions across all weight-loss thresholds and follow-up timepoints, with no changes in the direction or statistical interpretation of the findings (full comparisons are presented in Table S5 in Multimedia Appendix 1; post hoc power analyses are provided in S5 in Multimedia Appendix 1). Analyses in which weights were truncated at the first and 99th percentiles produced similarly consistent results.

##### Quantitative Bias Analysis for Unmeasured Confounding

For the day-180 ≥15% weight-loss outcome, the adjusted OR was 1.99, corresponding to an E-value of 3.39 for the point estimate and 1.83 for the lower confidence limit. For the day-180 ≥5% outcome, the adjusted OR was 2.22, corresponding to an E-value of 3.87 for the point estimate and 2.15 for the lower confidence limit.

These values indicate that unmeasured confounding of moderate-to-large magnitude could potentially explain the observed associations, particularly given plausible unmeasured differences in motivation, time availability, and digital literacy between participants who did and did not use app features. Accordingly, the results should be interpreted as associations rather than causal effects.

##### Kaplan-Meier Survival Curves

Kaplan-Meier curves were used as a complementary time-to-event analysis to examine time to first achievement of ≥5%, ≥10%, and ≥15% weight loss (Figure 1). Consistent with the logistic regression findings at prespecified timepoints, participants receiving the app+ medication intervention appeared to achieve weight-loss thresholds earlier and at higher estimated cumulative probabilities than those receiving medication alone. Separation of the curves was evident within the first 30‐60 days and generally widened over time, suggesting that app users experienced both earlier achievement of weight-loss milestones and a greater cumulative probability of reaching these thresholds during follow-up.

**Figure 1.**
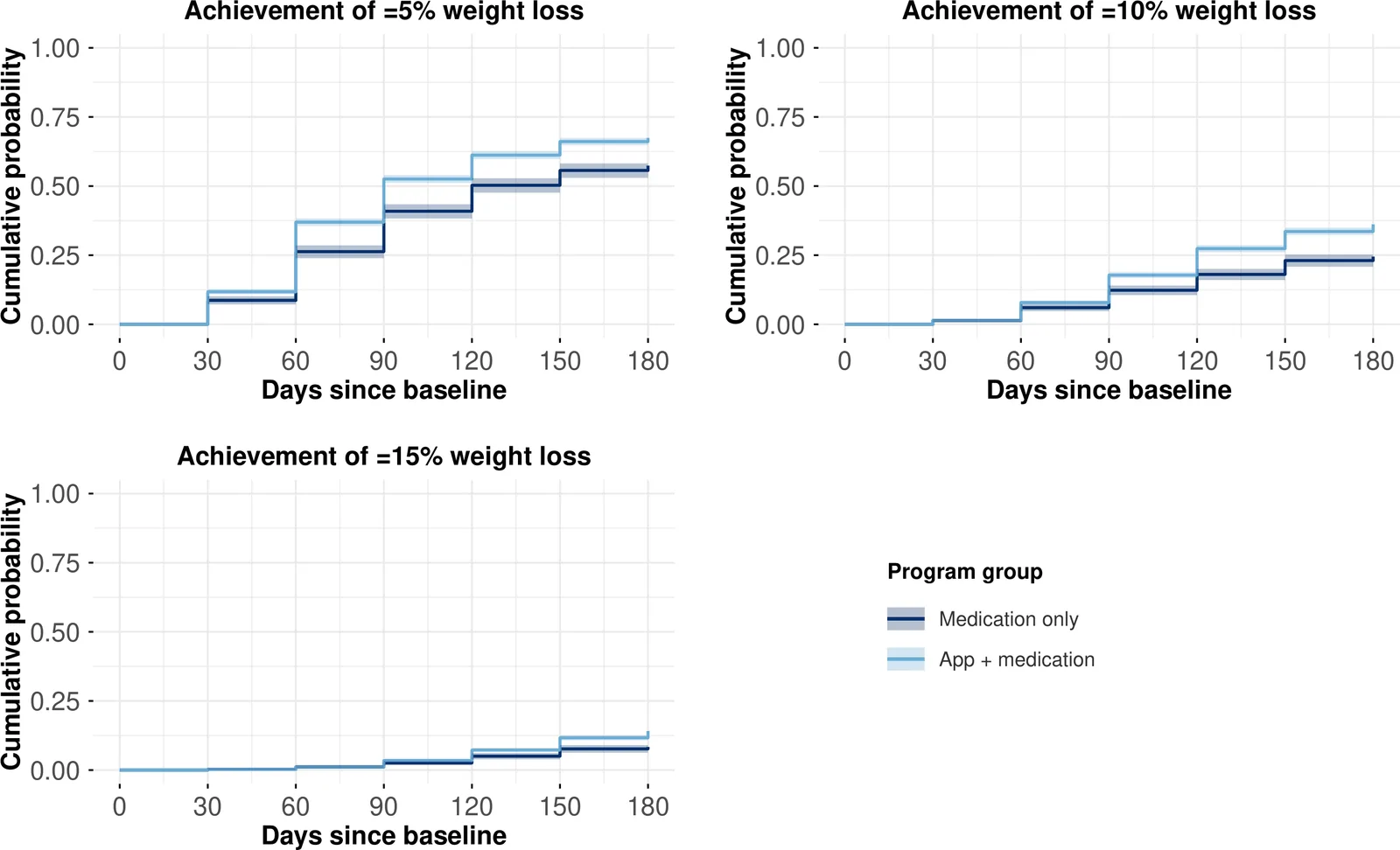
Kaplan-Meier curves showing the probability of achieving ≥5%, ≥10%, and ≥15% weight loss over 180 days among participants receiving either medication alone (the dark blue line) or the combined app+ medication intervention (the light blue line).

### Secondary Aim: App Features

Separate adjusted feature-specific models were fitted to examine which features show a clinically interpretable association with percentage weight-loss trajectory (available in Section S6 in Multimedia Appendix 1) resulted in the inclusion of calorie target setting, goal setting, nurse check-in completion, progress logging, recipe favoriting, and recipe screen viewing. In regard to quadratic predictors, only recipe-screen viewing showed evidence of improved fit (ΔAIC=−2.27) after inclusion of the quadratic interaction (results of model-fit analyses are provided in Section S7 in Multimedia Appendix 1); therefore, a recipe-screen-viewing-by-time² interaction was included in the final model, whereas the remaining feature-by-time associations were modeled as linear.

The final model included main effects for the abovementioned app features after centering and the interaction of their centered terms with linear time, with an additional quadratic interaction between centered recipe screen viewing and time. The model was adjusted for initial medication type, gender, ethnicity, age at first subscription, and baseline BMI. Interactions between linear time and medication type, and between linear time and gender, were also included as these added significantly to the model. A random intercept and random slope for phase were included for each participant to account for repeated measures and individual variation in weight-loss trajectories.

Written mathematically, the final model was specified as:


$$\begin{array}{rl} Y_{ij}=\; & \beta_{0}+\beta_{1}{\text{phase}}_{ij}+\beta_{2}{\text{phase}}_{ij}^{2}+\beta_{3}{\text{drug}}_{i}+\beta_{4}{\text{gender}}_{i}+\beta_{5}{\text{age}}_{i}\\ +\beta_{6}{\text{ethnicity}}_{i}+\beta_{7}{\text{baselineBMI}}_{i}+\beta_{8}{\text{calorie}}_{c_{i}}+\beta_{9}{\text{goal}}_{c_{i}}\\ +\beta_{10}{\text{nurse}}_{c_{i}}+\beta_{11}{\text{progress}}_{c_{i}}+\beta_{12}{\text{recipefav}}_{c_{i}}\\ +\beta_{13}{\text{recipeview}}_{c_{i}}\\ +\beta_{14}\left({\text{phase}}_{ij}\times {\text{calorie}}_{c_{i}}\right)\\ +\beta_{15}\left({\text{phase}}_{ij}\times {\text{goal}}_{c_{i}}\right)\\ +\beta_{16}\left({\text{phase}}_{ij}\times {\text{nurse}}_{c_{i}}\right)\\ +\beta_{17}\left({\text{phase}}_{ij}\times {\text{progress}}_{c_{i}}\right)\\ +\beta_{18}\left({\text{phase}}_{ij}\times {\text{recipefav}}_{c_{i}}\right)\\ +\beta_{19}\left({\text{phase}}_{ij}\times {\text{recipeview}}_{c_{i}}\right)\\ +\beta_{20}\left({\text{phase}}_{ij}^{2}\times {\text{recipeview}}_{c_{i}}\right)\\ +\beta_{21}\left({\text{phase}}_{ij}^{2}\times {\text{medicationtype}}_{i}\right)\\ +\beta_{22}\left({\text{phase}}_{ij}\times {\text{gender}}_{i}\right)\\ +b_{0i}+b_{1i}{\text{phase}}_{ij}+\varepsilon_{ij}. \end{array}$$

where Y_ij_ represents percentage weight loss (0‐100) for participant i at timepoint j, phase_ij_ is the study phase, and phase_ij_^2^ is the squared phase term. The app-feature variables represent centered binary indicators of whether participants used each feature. The term ${\text{b}}_{\text{0}\text{i}}$ represents the participant-specific random intercept, and ${\text{b}}_{\text{1}\text{i}}$ represents the participant-specific random slope for phase; together, ${\text{b}}_{\text{0}\text{i}}\text{+}{\text{b}}_{\text{1}\text{i}}{\text{phase}}_{\text{ij}}$ allow both overall percentage weight loss and rate of change over time to vary between participants. The residual error term is represented by $\varepsilon_{ij}$. The quadratic interaction was retained only for recipe screen viewing, rather than for all app features, to preserve model parsimony while allowing for the one nonlinear feature-specific trajectory that showed evidence of improved model fit.

Collinearity diagnostics did not indicate problematic multicollinearity in the final reduced model. VIFs for the app feature main effects and linear time interactions were low to moderate. The largest VIFs were observed for phase, phase², and the recipe-screen-viewing time interactions, which was expected given the inclusion of polynomial time terms and corresponding interaction terms. Adjusted VIFs were all below 3, suggesting that multicollinearity was unlikely to materially affect model interpretation.

Predictive checks indicated that model-predicted values closely resembled the observed distribution of percentage weight loss, suggesting that the model reproduced the overall outcome distribution reasonably well. Visual inspection of residual and random-effect diagnostic plots showed some deviation in the residual tails and possible influential observations; however, these did not indicate substantial model misspecification (model diagnostics are available in Section S9 in Multimedia Appendix 1). Participant-level influence was therefore examined using Cook’s distance. All Cook’s distance values were below the cut-off of 1.

In the reduced mixed-effects model (Table 5), percentage weight loss increased significantly over time (Figure 2), with a positive linear effect of time (β=3.04, SE 0.05; 2-tailed *t*_12550_=58.85, 95% CI 2.94-3.14; *P*<.001), and a negative quadratic effect (β=-.18, SE 0.01; *t*_10190_=-25.68, 95% CI -0.20 to ‐0.16; *P*<.001). This indicates that percentage weight loss increased over follow-up, but the rate of increase attenuated over time. Among the app features, progress logging was the clearest independent predictor of greater weight loss. Participants who logged progress had a greater increase in percentage weight loss over time (β=.26, SE 0.06; *t*_4336_=4.46, 95% CI 0.14-0.38; *P*<.001), and the quadratic interaction between recipe viewing and time was statistically significant (β=.03, SE 0.01; *t*_9988_=2.10, 95% CI 0.01-0.05; *P*=.04), suggesting that the association between recipe viewing and percentage weight-loss trajectory varied nonlinearly over follow-up (see S10 in Multimedia Appendix 1 for full results, including the covariates; a sensitivity analysis including dose-level as a predictor is also available in S11 in Multimedia Appendix 1; results are similar when dose level is included as a predictor).

**Table 5.** Linear mixed-effects model predicting percentage weight loss over time in the app+medication group^a^.

Predictor	Estimate	SE	*df*	t-statistics	*P* value	95% CI
Overall trajectory						
Intercept	−0.22	0.38	5800	−0.57	.57	−0.96 to 0.52
Time	3.04	0.05	12,550	58.85	<.001	2.94 to 3.14
Time²	−0.18	0.01	10,190	−25.68	<.001	−0.20 to −0.16
Baseline differences						
Set calorie target (centered)	0.14	0.16	4488	0.92	.36	−0.17 to 0.45
Set goal (centered)	0.20	0.18	4890	1.15	.25	−0.15 to 0.55
Nurse check-in completed (centered)	−0.07	0.11	4796	−0.67	.50	−0.29 to 0.15
Progress logged (centered)	0.07	0.16	5233	0.42	.68	−0.24 to 0.38
Recipe favorited (centered)	−0.56	0.26	4230	−2.14	.03	−1.07 to −0.05
Recipe viewed (centered)	0.15	0.17	8560	0.85	.39	−0.18 to 0.48
Interactions with time						
Set calorie target (centered)×time	−0.10	0.06	3734	−1.74	.08	−0.22 to 0.02
Set goal (centered)×time	0.04	0.07	4038	0.62	.54	−0.10 to 0.18
Nurse check-in completed (centered)×time	0.03	0.04	4001	0.66	.51	−0.05 to 0.11
Progress logged (centered)×time	0.26	0.06	4336	4.46	<.001	0.14 to 0.38
Recipe favourited (centered)×time	0.11	0.10	3469	1.18	.24	−0.09 to 0.31
Recipe viewed (centered)×time	−0.13	0.10	12,320	−1.27	.20	−0.33 to 0.07
Recipe viewed (centered)×time²	0.03	0.01	9988	2.10	.04	0.01 to 0.05

a Time refers to phase, coded from 0 to 6, corresponding to 0, 30, 60, 90, 120, 150, and 180 days. Time² refers to the squared phase term. App-feature variables were centred binary indicators. CIs are approximate Wald 95% CIs. Ethnicity estimates are interpreted relative to Caucasian participants.

**Figure 2.**
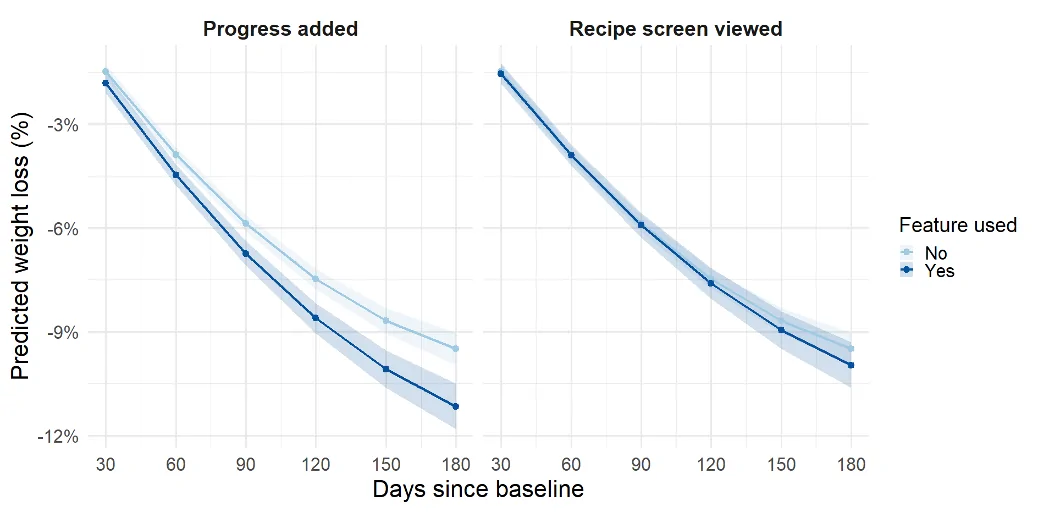
Predicted percentage weight-loss trajectories over 180 days by app feature use.

### IPCW Sensitivity Analyses

For the inverse probability weighted sensitivity analysis, the truncated combined weights ranged from 0.81 to 1.69, indicating that no observations received disproportionately large weights. The weighted sensitivity model produced estimates that were substantively unchanged from the unweighted model (Section S12 in Multimedia Appendix 1; post hoc power analyses are also available in Section S13 in Multimedia Appendix 1).

## Discussion

### Principal Results

Augmenting a direct-to-consumer GLP-1 program with the use of a smartphone app to support behavioral change was associated with more rapid weight loss, on average, than medication alone, and those using the app were more likely to achieve clinically significant weight loss than those not using the app. The added effect of app use was small relative to the total weight loss achieved through medication, but consistent with effect sizes observed in nonpharmacological interventions [18,40]. The only app features associated with weight loss were progress logging and recipe viewing. Importantly, participants reported no serious adverse outcomes.

Although the smartphone app incorporated several behavior change features aligned with evidence-based digital weight management strategies, only progress logging was associated with improved weight-loss trajectories over time, suggesting that active self-monitoring may play a meaningful role in sustaining behavior change. In contrast, goal setting and calorie target setting were not associated with weight-loss outcomes. Recipe viewing was not associated with early changes in weight loss but demonstrated a small positive quadratic effect, indicating that it may be associated with a potential delayed or increasing benefit over time.

These findings should be interpreted in light of how feature use was measured. Features were coded simply as “never used” versus “used at least once,” substantially limiting the ability to identify dose-response relationships. A single interaction with goal setting, recipe viewing, or dietitian consultation may have little behavioral effect, and binary coding obscures the frequency, intensity, and quality of engagement that often influence digital intervention outcomes [12,13,41].

Because app use was voluntary, observed associations may also reflect systematic differences between individuals who did and did not engage with the app. App users may have differed in motivation, readiness to change, digital literacy, time availability, or capacity for self-monitoring; conversely, app use may also have been greater among participants experiencing more difficulty or requiring additional support. Feature use was measured during follow-up, creating further potential for reverse causation whereby early weight-loss success encouraged continued engagement, while limited progress prompted greater app use. The available covariates did not capture these behavioral, socioeconomic, or time-varying factors. Although IPCW and E-value analyses suggest that the principal findings were reasonably robust under their respective assumptions, residual and time-varying confounding remain plausible. The results should therefore be interpreted as observational associations rather than causal effects.

### Comparison With Prior Work

The finding that those in the app+medication group tended to lose more weight than those in the medication-only group adds to a growing body of evidence that direct-to-consumer GLP-1 programs may be effective in reducing weight loss, and that augmentation with digital health tools may help to enhance this weight loss [5]. Compared to previous work, this study also found a lower dropout rate in the app+medication group [27,42]. However, participants self-selected into intervention groups, so the study was observational, not experimental; therefore, causational conclusions cannot be drawn.

The significant nonlinear time effects where early weight loss tapered over time are consistent with well-characterized physiological adaptations that oppose continued weight reduction, including reductions in resting energy expenditure, compensatory increases in appetite, and behavioral plateaus [43-45]. These processes have been consistently observed across both pharmacological and behavioral weight-loss interventions [46-49]. Clinically, this highlights the importance of continued follow-up and dose optimization and reinforces the value of modeling weight loss as a dynamic, nonlinear process.

Medication appeared to be a major driver of percentage weight loss, with both medication type and dose showing associated outcomes over time. This is consistent with clinical trials showing that incretin-based weight-management medications (eg, GLP-1 RAs and dual agonists) produce substantial and sustained reductions in body weight, often exceeding those achieved through behavioral programs alone [31,36,37].

Medication type was also a strong determinant of longitudinal outcomes. Although individuals receiving tirzepatide demonstrated lower initial weight loss, they experienced greater improvements over time compared with those receiving semaglutide. The stronger time interaction and more pronounced quadratic term suggest a trajectory of greater gains that subsequently plateau, broadly consistent with emerging evidence regarding the efficacy of dual agonist therapies [3,31,36]. These findings highlight the importance of considering treatment trajectories rather than only average differences at a single timepoint.

Progress logging was the app feature most clearly associated with greater weight loss, consistent with literature identifying self-monitoring as an important component of digital weight-loss interventions [10,11,41]. Self-regulatory models propose that monitoring progress provides feedback against goals, increases awareness of behavioral lapses, and can strengthen self-efficacy and adherence [11,29,50-56]. These mechanisms provide a plausible explanation for the observed association, although the binary measure of logging prevents conclusions about the amount or pattern of engagement required.

Recipe viewing was associated with weight loss insofar as weight loss tapered more quickly among those who did not view recipes. Recipe use may therefore support dietary choices that help sustain weight loss, although reverse causation is also possible if participants achieving greater weight loss were more attentive to meal selection.

The finding that only progress logging and recipe viewing predicted weight loss should not be interpreted as evidence that other behavioral tools are ineffective. Instead, it likely reflects measurement limitations in feature-use data and ceiling effects introduced by highly effective pharmacotherapy. Future research incorporating granular engagement metrics (eg, frequency, duration, streaks, and temporal patterns of feature use) will be better positioned to quantify the behavioral contribution of digital features alongside GLP-1 RA treatment.

These findings also have implications beyond the immediate clinical question of whether digital tools are associated with weight-loss outcomes during GLP-1 RA treatment. Direct-to-consumer GLP-1 RA platforms operate as hybrid systems: they combine medication prescribing, digital behavioral support, self-monitoring infrastructure, and consumer-directed health service delivery [5,57]. Such programs sit within an expanding digital health and food environment in which online interfaces can influence exposure, choice, and behavior. Policy commentary has argued that obesity and food policy increasingly needs to consider “the screen as well as the street”behavior [58]. As digital-pharmacological programs expand, evaluation should therefore consider not only medication safety and clinical outcomes, but also how app design, engagement strategies, and behavioral content influence patient behavior [59,60].

Digital behavioral support may also offer scalability advantages because features such as self-monitoring and personalized feedback can be delivered to large populations with relatively little additional workforce burden. Consequently, even modest incremental benefits could be meaningful when delivered alongside effective pharmacotherapy at scale.

### Limitations

A key limitation of this study is the self-selection of participants into the app+medication group. Although this reflects real-world uptake, differences in outcomes may partly reflect preexisting differences between groups. For example, app users may have been more motivated to lose weight, although app uptake could also have been greater among people requiring additional support. Johnson et al [25] found that individuals with a higher burden of baseline comorbidities were more engaged with digital augmentation of a GLP-1 weight-loss intervention. Only basic participant characteristics were available in the dataset and socioeconomic status and geographic locations were unavailable. These factors may be associated with digital access, app engagement, access to health-promoting resources, and weight-loss outcomes; consequently, residual confounding by socioeconomic or geographic differences cannot be excluded.

Although the regression models adjusted for age, gender, ethnicity, baseline BMI, and initial medication type, they could not account for factors such as readiness to change, health and digital literacy, available time, socioeconomic circumstances, or access to social support. These factors were not available in the dataset and could plausibly affect both engagement with the app and subsequent weight loss. Consequently, the adjusted estimates should not be interpreted as isolating the causal effect of the app. Greater motivation among app users may also have been sustained by app use, and the lower cancellation rate in the app+medication group is consistent with greater ongoing engagement. Nonetheless, determining whether the app itself causes additional weight loss would require a controlled experimental study.

A further limitation is the measurement of app engagement. No information was collected on how frequently, or for how long, each feature was used, preventing assessment of “dose-response” relationships despite previous evidence suggesting that intensity of engagement may matter [11-13]. A participant who used a feature once was treated identically to someone who used it repeatedly. Feature availability or one-off contact therefore should not be interpreted as meaningful behavioral engagement. Future studies should collect measures of frequency, recency, duration, cumulative exposure, and temporal patterns of use behavior.

Weight outcomes were also self-reported using participants’ own scales rather than measured under standardized research conditions. Self-reported weight generally shows high agreement with measured weight, including in epidemiological and digital health studies [61]. One web-based validation study reported a correlation of 0.98, although self-reported weight was underestimated by an average of 1.2 kg and underreporting increased at higher body weight [62]. Reporting bias may also vary by BMI, age, sex, and other participant characteristics [61,63]. Both groups reported weight through the same platform, reducing concern about measurement-mode differences between groups. Moreover, because this was a longitudinal study, weight loss was calculated by comparing each participant’s later weight with their own baseline weight, meaning that stable within-person reporting tendencies may have had less impact on estimated weight change than on absolute weight. However, differential reporting bias cannot be excluded; for example, app users may have weighed themselves more frequently or reported weight differently from nonusers.

Informative dropout remains a potential limitation. Although the IPCW sensitivity analysis produced results similar to the primary model, outcomes were unobservable after subscription cancellation. Unmeasured factors associated with both cancellation and weight loss therefore could have biased later follow-up estimates, particularly at 120 and 180 days. Weighting adjusted for measured predictors of observation and censoring, but cannot eliminate bias from unmeasured predictors.

Finally, this study was funded by Hairmop (Mosh), who also collected and provided the underlying data and suggested the initial operational definition of the app+medication group. Participants were classified as app+medication if they had any recorded use because the primary research question concerned whether any use of the app, alongside medication, was associated with weight-loss outcomes. Alternative thresholds based on frequency or intensity would have been arbitrary and could not be examined because more detailed engagement data were unavailable.

The researchers had independent access to the analytic dataset and retained responsibility for data cleaning, model specification, sensitivity analyses, interpretation, and reporting. The sponsor did not restrict the analyses that could be conducted or require approval of the findings. Nevertheless, sponsor funding, sponsor-provided data, and evaluation of the sponsor’s own program represent potential sources of bias. In addition, exposure classification was constrained by the app-use variables available within the sponsor’s data systems. The findings should therefore be interpreted as observational associations rather than causal effects, although their consistency across alternative exposure definitions and sensitivity analyses provides some reassurance regarding robustness.

### Conclusions

Participants who used a smartphone app alongside their direct-to-consumer GLP-1 experienced greater average weight loss and were more likely to achieve clinically significant milestones than those relying on medication alone. Although the incremental effects were modest relative to those of pharmacotherapy, consistent with expectations when augmenting highly potent medications, they were statistically robust and clinically meaningful.

The findings support the potential for digital-pharmacologic hybrid models for scalable weight-management care. Integrating behavioral tools with GLP-1 treatment may enhance weight outcomes and support sustained engagement. However, because this was an observational study with self-selected app use, causational conclusions cannot be drawn.

## Supplementary material

10.2196/100728 Multimedia Appendix 1 Statistical analyses, including model selection, additional regression results, sensitivity analyses, inverse probability-of-censoring weighted analyses, and post hoc power analyses.

## Abbreviations

AIC: Akaike information criterion
BCT: behavior-change technique
GLP-1 RA: glucagon-like peptide-1 receptor agonist
HREC: Human Research Ethics Committee
IPCW: inverse probability-of-censoring weighted
OR: odds ratio
VIF: variance inflation factor
