# Supplementary material for: Digital Augmentation of Glucagon-Like Peptide-1 for Obesity Management: Retrospective Study
Source: J Med Internet Res. 2026 Nov 3;28:e100728. doi: 10.2196/100728 (PMC13730510; doi:10.2196/100728)
Supplement: Supplementary file 1 [file jmir-v28-e100728-s001.docx]

## S1: Results comparing participants who actively cancelled to those who did not

Those who actively cancelled their subscription were compared to those retained for analyses (see Table S1). Table S1 shows that all standardised mean differences on all characteristics were negligible (<.10) or small (<0.20).

#### Table S1. Comparison of participants who cancelled their Mosh subscription with those who did not cancel their Mosh subscription.

| **Characteristic** | **N** | **Overall** | **Did not cancel** | **Cancelled** | **SMD (95% CI)** |
| --- | --- | --- | --- | --- | --- |
| **Baseline weight, kg** | 8148 | 96.92 (18.9) | 95.38 (17.97) | 97.84 (19.38) | 0.132 (0.083, 0.176) |
| **Baseline BMI, kg/m²** | 8148 | 34.03 (5.38) | 33.63 (4.88) | 34.27 (5.64) | 0.122 (0.077, 0.165) |
| **Age** | 8143 | 38.05 (10.39) | 38.93 (10.13) | 37.53 (10.51) | -0.135 (-0.18, -0.094) |
| **Gender** | 8148 |  |  |  | 0.046 (0.003, 0.091) |
| female |  | 5755 (70.6%) | 2195 (71.9%) | 3560 (69.8%) |  |
| male |  | 2393 (29.4%) | 856 (28.1%) | 1537 (30.2%) |  |
| **Ethnicity** | 8146 |  |  |  | 0.059 (0.013, 0.100) |
| Aboriginal or Torres Strait Islander |  | 235 (2.9%) | 81 (2.7%) | 154 (3%) |  |
| African |  | 96 (1.2%) | 36 (1.2%) | 60 (1.2%) |  |
| Asian |  | 420 (5.2%) | 136 (4.5%) | 284 (5.6%) |  |
| Caucasian |  | 6279 (77.1%) | 2394 (78.5%) | 3885 (76.2%) |  |
| Latino/Hispanic |  | 133 (1.6%) | 48 (1.6%) | 85 (1.7%) |  |
| Middle Eastern |  | 181 (2.2%) | 55 (1.8%) | 126 (2.5%) |  |
| Not listed |  | 626 (7.7%) | 250 (8.2%) | 376 (7.4%) |  |
| Pacific Islander or Maori |  | 176 (2.2%) | 50 (1.6%) | 126 (2.5%) |  |
| **Program** |  |  |  |  | 0.407 (0.361, 0.453) |
| Medication only |  | 2683 (32.9%) | 654 (24.4) | 2029 (75.6) |  |
| App + medication |  | 5465 (67.1%) | 2397 (43.9) | 3068 (56.1) |  |

^1^SMD = standardised mean difference – this describes the size of the difference in standardised deviation units; SMD < 0.10 negligible imbalance, 0.10–0.20 small imbalance, > 0.20 potentially meaningful imbalance

^2^ CI = Confidence Interval

## S2: Weight loss data for program by gender

#### Table S2: Weight loss data for program by gender

| **Day** | **Medication only** | | | | | | **Program + medication** | | | | | |
| --- | --- | --- | --- | --- | --- | --- | --- | --- | --- | --- | --- | --- |
|  | **Female** | | | **Male** | | | **Female** | | **Male** | | | |
|  | n | Weight, mean (SD) | Percent weight loss, mean (SD) | n | Weight, mean (SD) | Percent weight loss, mean (SD) | n | Weight, mean (SD) | Percent weight loss, mean (SD) | n | Weight, mean (SD) | Percent weight loss, mean (SD) |
| 0 | 838 | 91.11 (15.75) |  | 559 | 108.23 (18.67) |  | 3,506 | 92.53 (16.40) |  | 1,273 | 108.42 (18.58) |  |
| 30 | 420 | 90.21 (16.82) | 2.24 (3.57) | 302 | 105.35 (19.26) | 2.14 (3.36) | 2,523 | 91.00 (16.58) | 2.39 (3.24) | 864 | 105.71 (18.64) | 2.53 (3.05) |
| 60 | 434 | 86.43 (16.26) | 4.38 (4.57) | 322 | 103.67 (18.12) | 4.44 (3.86) | 2,482 | 88.20 (16.40) | 4.86 (4.03) | 871 | 103.33 (17.47) | 5.02 (3.95) |
| 90 | 353 | 86.09 (16.07) | 6.42 (4.96) | 238 | 103.32 (16.99) | 5.71 (4.22) | 1,970 | 85.84 (16.00) | 7.15 (4.81) | 726 | 101.91 (18.61) | 6.90 (4.51) |
| 120 | 290 | 83.45 (14.26) | 8.34 (4.96) | 153 | 99.81 (17.91) | 6.74 (4.53) | 1,598 | 83.97 (14.97) | 8.87 (5.39) | 589 | 100.11 (19.31) | 8.66 (5.08) |
| 150 | 209 | 81.03 (16.26) | 10.04 (6.61) | 143 | 100.15 (18.15) | 8.27 (5.21) | 1,317 | 82.12 (15.32) | 10.74 (5.99) | 479 | 98.65 (18.19) | 9.93 (5.51) |
| 180 | 86 | 82.36 (16.13) | 9.13 (6.92) | 44 | 95.35 (14.89) | 8.70 (5.83) | 645 | 80.71 (14.08) | 11.93 (6.41) | 236 | 96.97 (18.09) | 10.76 (6.33) |

## S3: Full results for logistic regressions predicting the likelihood of achieving clinically significant weight-loss thresholds from program group adjusting for covariates

Table S3 provides the full results, including covariates, for the logistic regressions predicting the likelihood of achieving various clinically significant weight-loss thresholds on day 60, 120 and 180, adjusting for age, gender, ethnicity, initial medication type and baseline BMI.

##### Table S3. Full adjusted logistic regression models predicting clinically significant weight loss

| **Threshold** | **Timepoint** | **Model n** | **Predictor** | **Adjusted odds ratio (95% CI)** | **z** | **P value** |
| --- | --- | --- | --- | --- | --- | --- |
| ≥5% | Day 60 | 4,063 |  |  |  |  |
|  |  |  | App + medication | 1.40 (1.19, 1.66) | 3.98 | <.001 |
|  |  |  | Age | 0.99 (0.98, 1.00) | −2.95 | .003 |
|  |  |  | Male gender | 1.09 (0.95, 1.26) | 1.22 | .222 |
|  |  |  | Baseline BMI | 0.97 (0.95, 0.98) | −5.33 | <.001 |
|  |  |  | Medication type | 2.16 (1.87, 2.49) | 10.59 | <.001 |
|  |  |  | Aboriginal or Torres Strait Islander ethnicity | 1.23 (0.83, 1.82) | 1.03 | .304 |
|  |  |  | African ethnicity | 1.04 (0.55, 1.95) | 0.11 | .913 |
|  |  |  | Asian ethnicity | 0.58 (0.42, 0.79) | −3.43 | <.001 |
|  |  |  | Latino/Hispanic ethnicity | 0.63 (0.36, 1.07) | −1.70 | .089 |
|  |  |  | Middle Eastern ethnicity | 1.30 (0.82, 2.07) | 1.11 | .267 |
|  |  |  | Ethnicity not listed | 1.03 (0.81, 1.31) | 0.27 | .784 |
|  |  |  | Pacific Islander or Māori ethnicity | 0.82 (0.51, 1.29) | −0.86 | .388 |
| ≥10% | Day 60 | 4,063 |  |  |  |  |
|  |  |  | App + medication | 1.05 (0.80, 1.39) | 0.33 | .739 |
|  |  |  | Age | 0.98 (0.97, 0.99) | −3.47 | <.001 |
|  |  |  | Male gender | 1.13 (0.89, 1.42) | 1.01 | .315 |
|  |  |  | Baseline BMI | 0.97 (0.95, 1.00) | −2.38 | .017 |
|  |  |  | Medication type | 2.63 (2.12, 3.25) | 8.84 | <.001 |
|  |  |  | Aboriginal or Torres Strait Islander ethnicity | 0.81 (0.37, 1.55) | −0.59 | .556 |
|  |  |  | African ethnicity | 0.77 (0.23, 1.97) | −0.49 | .627 |
|  |  |  | Asian ethnicity | 0.87 (0.51, 1.41) | −0.52 | .601 |
|  |  |  | Latino/Hispanic ethnicity | 0.88 (0.33, 1.93) | −0.29 | .773 |
|  |  |  | Middle Eastern ethnicity | 0.81 (0.35, 1.62) | −0.56 | .578 |
|  |  |  | Ethnicity not listed | 1.17 (0.79, 1.69) | 0.83 | .408 |
|  |  |  | Pacific Islander or Māori ethnicity | 0.57 (0.20, 1.29) | −1.21 | .228 |
| ≥15% | Day 60 | 4,063 |  |  |  |  |
|  |  |  | App + medication | 0.70 (0.36, 1.47) | −1.00 | .318 |
|  |  |  | Age | 0.96 (0.93, 0.99) | −2.37 | .018 |
|  |  |  | Male gender | 0.92 (0.45, 1.76) | −0.25 | .801 |
|  |  |  | Baseline BMI | 0.94 (0.87, 1.00) | −1.76 | .078 |
|  |  |  | Medication type | 1.89 (1.01, 3.45) | 2.05 | .041 |
|  |  |  | Aboriginal or Torres Strait Islander ethnicity | 0.88 (0.05, 4.19) | −0.13 | .898 |
|  |  |  | African ethnicity | 0.00 (0.00, 408,321.29) | −0.02 | .983 |
|  |  |  | Asian ethnicity | 1.31 (0.31, 3.76) | 0.43 | .665 |
|  |  |  | Latino/Hispanic ethnicity | 1.51 (0.08, 7.32) | 0.40 | .687 |
|  |  |  | Middle Eastern ethnicity | 3.03 (0.71, 9.00) | 1.77 | .077 |
|  |  |  | Ethnicity not listed | 1.72 (0.58, 4.09) | 1.11 | .267 |
|  |  |  | Pacific Islander or Māori ethnicity | 1.25 (0.07, 6.00) | 0.21 | .830 |
| ≥5% | Day 120 | 2,604 |  |  |  |  |
|  |  |  | App + medication | 1.38 (1.09, 1.75) | 2.71 | .007 |
|  |  |  | Age | 1.00 (0.99, 1.01) | −0.01 | .991 |
|  |  |  | Male gender | 0.95 (0.78, 1.17) | −0.47 | .636 |
|  |  |  | Baseline BMI | 0.97 (0.96, 0.99) | −3.01 | .003 |
|  |  |  | Medication type | 1.89 (1.51, 2.36) | 5.57 | <.001 |
|  |  |  | Aboriginal or Torres Strait Islander ethnicity | 0.81 (0.48, 1.46) | −0.72 | .470 |
|  |  |  | African ethnicity | 1.42 (0.47, 6.15) | 0.55 | .580 |
|  |  |  | Asian ethnicity | 0.95 (0.61, 1.53) | −0.21 | .835 |
|  |  |  | Latino/Hispanic ethnicity | 0.70 (0.32, 1.71) | −0.84 | .401 |
|  |  |  | Middle Eastern ethnicity | 1.40 (0.65, 3.47) | 0.79 | .431 |
|  |  |  | Ethnicity not listed | 1.10 (0.78, 1.58) | 0.51 | .607 |
|  |  |  | Pacific Islander or Māori ethnicity | 0.79 (0.43, 1.53) | −0.75 | .453 |
| ≥10% | Day 120 | 2,604 |  |  |  |  |
|  |  |  | App + medication | 1.67 (1.33, 2.10) | 4.42 | <.001 |
|  |  |  | Age | 0.99 (0.98, 0.99) | −3.58 | <.001 |
|  |  |  | Male gender | 0.76 (0.63, 0.91) | −2.99 | .003 |
|  |  |  | Baseline BMI | 0.98 (0.96, 1.00) | −2.33 | .020 |
|  |  |  | Medication type | 2.02 (1.70, 2.41) | 7.92 | <.001 |
|  |  |  | Aboriginal or Torres Strait Islander ethnicity | 0.70 (0.40, 1.17) | −1.35 | .178 |
|  |  |  | African ethnicity | 1.10 (0.45, 2.71) | 0.21 | .833 |
|  |  |  | Asian ethnicity | 0.72 (0.47, 1.08) | −1.56 | .119 |
|  |  |  | Latino/Hispanic ethnicity | 0.79 (0.36, 1.70) | −0.58 | .561 |
|  |  |  | Middle Eastern ethnicity | 1.10 (0.58, 2.06) | 0.29 | .774 |
|  |  |  | Ethnicity not listed | 1.08 (0.80, 1.46) | 0.51 | .612 |
|  |  |  | Pacific Islander or Māori ethnicity | 0.57 (0.30, 1.02) | −1.83 | .068 |
| ≥15% | Day 120 | 2,604 |  |  |  |  |
|  |  |  | App + medication | 1.27 (0.89, 1.84) | 1.28 | .199 |
|  |  |  | Age | 0.98 (0.96, 0.99) | −3.55 | <.001 |
|  |  |  | Male gender | 0.74 (0.54, 0.99) | −1.98 | .047 |
|  |  |  | Baseline BMI | 0.98 (0.95, 1.01) | −1.44 | .150 |
|  |  |  | Medication type | 2.51 (1.95, 3.24) | 7.09 | <.001 |
|  |  |  | Aboriginal or Torres Strait Islander ethnicity | 0.60 (0.21, 1.39) | −1.07 | .285 |
|  |  |  | African ethnicity | 0.51 (0.08, 1.81) | −0.89 | .372 |
|  |  |  | Asian ethnicity | 0.72 (0.34, 1.34) | −0.97 | .331 |
|  |  |  | Latino/Hispanic ethnicity | 1.81 (0.65, 4.36) | 1.24 | .214 |
|  |  |  | Middle Eastern ethnicity | 0.49 (0.12, 1.40) | −1.16 | .247 |
|  |  |  | Ethnicity not listed | 1.34 (0.86, 2.03) | 1.34 | .181 |
|  |  |  | Pacific Islander or Māori ethnicity | 0.55 (0.16, 1.38) | −1.13 | .258 |
| ≥5% | Day 180 | 1,001 |  |  |  |  |
|  |  |  | App + medication | 2.22 (1.40, 3.45) | 3.46 | <.001 |
|  |  |  | Age | 1.02 (1.00, 1.04) | 2.40 | .016 |
|  |  |  | Male gender | 0.63 (0.44, 0.92) | −2.41 | .016 |
|  |  |  | Baseline BMI | 0.99 (0.96, 1.03) | −0.54 | .591 |
|  |  |  | Medication type | 1.78 (1.17, 2.77) | 2.63 | .009 |
|  |  |  | Aboriginal or Torres Strait Islander ethnicity | 0.86 (0.31, 3.09) | −0.26 | .798 |
|  |  |  | African ethnicity | 0.95 (0.25, 6.26) | −0.07 | .947 |
|  |  |  | Asian ethnicity | 0.56 (0.28, 1.21) | −1.57 | .117 |
|  |  |  | Latino/Hispanic ethnicity | 1.88 (0.35, 34.83) | 0.59 | .553 |
|  |  |  | Middle Eastern ethnicity | 1.03 (0.26, 6.88) | 0.04 | .966 |
|  |  |  | Ethnicity not listed | 1.04 (0.53, 2.24) | 0.11 | .913 |
|  |  |  | Pacific Islander or Māori ethnicity | 0.41 (0.12, 1.59) | −1.41 | .160 |
| ≥10% | Day 180 | 1,001 |  |  |  |  |
|  |  |  | App + medication | 1.97 (1.35, 2.90) | 3.48 | <.001 |
|  |  |  | Age | 1.00 (0.98, 1.01) | −0.50 | .614 |
|  |  |  | Male gender | 0.60 (0.45, 0.79) | −3.55 | <.001 |
|  |  |  | Baseline BMI | 1.00 (0.97, 1.02) | −0.32 | .749 |
|  |  |  | Medication type | 1.73 (1.30, 2.32) | 3.71 | <.001 |
|  |  |  | Aboriginal or Torres Strait Islander ethnicity | 0.78 (0.32, 1.94) | −0.54 | .591 |
|  |  |  | African ethnicity | 0.42 (0.13, 1.26) | −1.54 | .123 |
|  |  |  | Asian ethnicity | 0.60 (0.32, 1.13) | −1.58 | .113 |
|  |  |  | Latino/Hispanic ethnicity | 0.81 (0.24, 2.87) | −0.35 | .728 |
|  |  |  | Middle Eastern ethnicity | 1.58 (0.50, 6.00) | 0.74 | .458 |
|  |  |  | Ethnicity not listed | 1.33 (0.80, 2.26) | 1.08 | .281 |
|  |  |  | Pacific Islander or Māori ethnicity | 0.96 (0.29, 3.35) | −0.07 | .943 |
| ≥15% | Day 180 | 1,001 |  |  |  |  |
|  |  |  | App + medication | 1.99 (1.26, 3.29) | 2.82 | .005 |
|  |  |  | Age | 0.99 (0.97, 1.00) | −1.85 | .065 |
|  |  |  | Male gender | 0.75 (0.54, 1.04) | −1.72 | .086 |
|  |  |  | Baseline BMI | 0.99 (0.96, 1.02) | −0.81 | .419 |
|  |  |  | Medication type | 1.76 (1.31, 2.36) | 3.73 | <.001 |
|  |  |  | Aboriginal or Torres Strait Islander ethnicity | 0.94 (0.33, 2.39) | −0.13 | .899 |
|  |  |  | African ethnicity | 0.78 (0.21, 2.42) | −0.42 | .677 |
|  |  |  | Asian ethnicity | 0.47 (0.20, 0.98) | −1.88 | .060 |
|  |  |  | Latino/Hispanic ethnicity | 0.81 (0.17, 2.89) | −0.30 | .761 |
|  |  |  | Middle Eastern ethnicity | 0.67 (0.15, 2.28) | −0.59 | .557 |
|  |  |  | Ethnicity not listed | 1.00 (0.57, 1.69) | 0.00 | .997 |
|  |  |  | Pacific Islander or Māori ethnicity | 0.19 (0.01, 1.03) | −1.55 | .121 |

*Note.* Odds ratios are mutually adjusted for program group, age, gender, ethnicity, baseline BMI, and initial medication type. The reference groups were medication only for program group, female for gender, and Caucasian for ethnicity. Model *n* represents the complete-case analytic sample. The medication-type odds ratio compares the category coded 1 with the reference category coded 0; the medication names should be inserted after confirming the coding of drug_initial. The extremely imprecise estimate for African ethnicity in the day-60 ≥15% model reflects sparse events and separation and should not be interpreted substantively.

Sensitivity analyses adjusted for final medication dose level for each logistic regression. Results are shown in Table S4,

##### Table S4a: Sensitivity analysis: Comparing outcomes of logistic regression models predicting the likelihood of achieving various clinical weight-loss thresholds at 60, 120 and 180 days, statistically adjusting for gender, baseline BMI, ethnicity and medication type, with and without final dose level as a covariate

| **Outcome** | **Original model*** | | | **Results with final dose level included as a predictor** | | |
| --- | --- | --- | --- | --- | --- | --- |
|  | **ORs** | **P** | **AIC** | **ORs** | **P** | **AIC** |
| **60-day** |  |  |  |  |  |  |
| ≥5% loss | 1.40 | <.001 | 5,456.0 | 1.41 | <.001 | 5,452.6 |
| ≥10% loss | 1.05 | .739 | 2,532.4 | 1.06 | .681 | 2,524.8 |
| ≥15% loss | 0.70 | .318 | 501.6 | 0.71 | .340 | 500.3 |
| **120-day** |  |  |  |  |  |  |
| ≥5% loss | 1.38 | .007 | 2,774.7 | 1.37 | .008 | 2,771.2 |
| ≥10% loss | 1.67 | <.001 | 3,392.7 | 1.68 | <.001 | 3,391.2 |
| ≥15% loss | 1.27 | .199 | 1,732.2 | 1.29 | .170 | 1,715.0 |
| **180-day** |  |  |  |  |  |  |
| ≥5% loss | 2.22 | <.001 | 840.1 | 2.09 | .002 | 824.3 |
| ≥10% loss | 1.97 | <.001 | 1,336.6 | 1.92 | <.001 | 1,331.6 |
| ≥15% loss | 1.99 | .005 | 1,186.6 | 1.97 | .006 | 1,187.3 |

*Note.* The original models were adjusted for program group, age, gender, ethnicity, baseline BMI, and initial medication type. The dose-adjusted models additionally included final dose level. Odds ratios for program group compare the app + medication group with the medication-only group. Dose-level odds ratios represent the change in the odds of reaching the relevant weight-loss threshold for each one-unit increase in final dose level.

*Original model = model without the final dose level included as a covariate

##### Table S4b: OR and p-values for dose-level in the models predicting the likelihood of achieving various clinically significant thresholds at day 60, 120 and 180, including dose-level as a predictor

| **Outcome** | **For dose-level in the dose-level model** |  |
| --- | --- | --- |
|  | **OR** | **P** |
| **60-day** |  |  |
| ≥5% loss | 0.92 | .020 |
| ≥10% loss | 0.83 | .002 |
| ≥15% loss | 0.73 | .071 |
| **120-day** |  |  |
| ≥5% loss | 1.12 | .020 |
| ≥10% loss | 0.92 | .063 |
| ≥15% loss | 0.74 | <.001 |
| **180-day** |  |  |
| ≥5% loss | 1.45 | <.001 |
| ≥10% loss | 1.19 | .009 |
| ≥15% loss | 1.08 | .259 |

Sensitivity analyses additionally adjusting for final medication dose produced results that were substantively consistent with the primary models. The estimated association between app + medication participation and clinically significant weight loss changed only minimally across all thresholds and timepoints, and the statistical interpretation of each program effect remained unchanged. The app + medication group continued to have significantly greater odds of achieving at least 5% weight loss at days 60, 120, and 180; at least 10% weight loss at days 120 and 180; and at least 15% weight loss at day 180. Associations remained non-significant for the 10% and 15% thresholds at day 60 and the 15% threshold at day 120. Model fit generally improved slightly after final dose level was added, with lower AIC values for eight of the nine models, although the magnitude of improvement varied. These findings indicate that the observed association between program participation and weight-loss outcomes was not explained by differences in final medication dose.

## S4: Full results for IPW sensitivity analyses for logistic regressions predicting the likelihood of achieving various clinically significant weight-loss thresholds

Full results of the IPW sensitivity analyses compared to the original results for the logistic regressions are available in Table S4.

#### Table S5. Comparison of OR (CI), z and p for the original analysis versus the IPW sensitivity analysis for each logistic regression predicting various clinically significant weight-loss thresholds at 60, 120 and 180 days

| **Threshold** | **Day** | **n** | **Original OR (95% CI)** | **IPCW OR (95% CI)** | **Original z** | **IPCW z** | **Original P** | **IPCW P** |
| --- | --- | --- | --- | --- | --- | --- | --- | --- |
| 5% | 60 | 4,063 | 1.40 (1.19, 1.66) | 1.42 (1.20, 1.68) | 3.98 | 4.05 | <.001 | <.001 |
| 5% | 120 | 2,604 | 1.38 (1.09, 1.75) | 1.39 (1.10, 1.75) | 2.71 | 2.72 | .007 | .007 |
| 5% | 180 | 1,001 | 2.22 (1.40, 3.45) | 2.40 (1.53, 3.78) | 3.46 | 3.80 | <.001 | <.001 |
| 10% | 60 | 4,063 | 1.05 (0.80, 1.39) | 1.08 (0.81, 1.43) | 0.33 | 0.51 | .739 | .613 |
| 10% | 120 | 2,604 | 1.67 (1.33, 2.10) | 1.69 (1.34, 2.12) | 4.42 | 4.42 | <.001 | <.001 |
| 10% | 180 | 1,001 | 1.97 (1.35, 2.90) | 2.08 (1.41, 3.06) | 3.48 | 3.71 | <.001 | <.001 |
| 15% | 60 | 4,063 | 0.70 (0.36, 1.47) | 0.72 (0.36, 1.45) | −1.00 | −0.92 | .318 | .359 |
| 15% | 120 | 2,604 | 1.27 (0.89, 1.84) | 1.24 (0.85, 1.80) | 1.28 | 1.13 | .199 | .257 |
| 15% | 180 | 1,001 | 1.99 (1.26, 3.29) | 2.03 (1.25, 3.31) | 2.82 | 2.85 | .005 | .004 |

## S5: Post-hoc power analysis for logistic regressions

Post-hoc sensitivity analyses were conducted to characterise the magnitude of effects that could be detected by the realised analytic samples. For each logistic-regression model, the standard error of the log odds ratio was derived from the reported program-group coefficient and Wald *z* statistic. Approximate power for the observed odds ratio was then calculated using a two-sided α level of .05. The minimum detectable odds ratio for 80% power was estimated as $\exp\left[ \left( z_{.975}+z_{.80} \right)\times SE_{\log\left( OR \right)} \right]$, and the equivalent protective association was calculated as its reciprocal. These calculations describe the sensitivity of the completed analyses and should not be interpreted as prospective sample-size calculations.

#### Table S6: Power analysis for logistic regressions showing power for observed odds ratios and detectable odds ratios given the sample size using Wald-test calculations

| **Threshold** | **Day** | **Model n** | **Observed OR** | **Approximate observed power** | **Smallest OR above 1 detectable with 80% power** | **Equivalent OR below 1** |
| --- | --- | --- | --- | --- | --- | --- |
| ≥5% | 60 | 4,063 | 1.40 | 97.8% | 1.27 | 0.79 |
| ≥5% | 120 | 2,604 | 1.38 | 77.3% | 1.40 | 0.72 |
| ≥5% | 180 | 1,001 | 2.22 | 93.3% | 1.91 | 0.52 |
| ≥10% | 60 | 4,063 | 1.05 | 6.3% | 1.51 | 0.66 |
| ≥10% | 120 | 2,604 | 1.67 | 99.3% | 1.38 | 0.72 |
| ≥10% | 180 | 1,001 | 1.97 | 93.6% | 1.73 | 0.58 |
| ≥15% | 60 | 4,063 | 0.70 | 17.0% | 2.72 | 0.37 |
| ≥15% | 120 | 2,604 | 1.27 | 24.9% | 1.69 | 0.59 |
| ≥15% | 180 | 1,001 | 1.99 | 80.5% | 1.98 | 0.50 |

Approximate power varied substantially across outcomes. The analyses had high power for the observed ≥5% weight-loss associations at days 60 and 180, the ≥10% associations at days 120 and 180, and the ≥15% association at day 180. Power for the day-120 ≥5% association was approximately 77%, slightly below the conventional 80% benchmark. The analyses had limited power for the small or null associations at the day-60 ≥10% threshold and the day-60 and day-120 ≥15% thresholds. Accordingly, nonsignificant findings for these outcomes should not be interpreted as evidence that smaller associations were absent.

## S6: Results of separate mixed-effect models for each app feature

Feature-specific mixed-effects models were estimated to examine the association between each app feature and percentage weight-loss trajectory over time (see Table S7). Each app feature was examined in a separate adjusted model that included linear and quadratic effects of time, the app-feature main effect, and the interaction between the app feature and linear time, with adjustment for medication type, gender, age, ethnicity, and baseline BMI. Random intercepts and random slopes for time were included for each participant to account for repeated measures and individual variation in weight-loss trajectories. The estimates shown represent unstandardised coefficients for the app feature × time interaction; therefore, they indicate the difference in rate of percentage weight loss over time between participants who used the feature and those who did not.

#### Table S7: Results of each separate app-feature mixed-effect model

| **App feature** | **b** | **std.error** | **t** | **df** | **p.value** |
| --- | --- | --- | --- | --- | --- |
| **Set calorie target** | 0.126 | 0.0422 | 2.98 | 3930 | <.001 |
| **Dietitian consult** | -0.010 | 0.0716 | -0.135 | 3978 | .893 |
| **Set goal** | 0.219 | 0.0417 | 5.24 | 4171 | <.001 |
| **Log meal** | 0.134 | 0.0877 | 1.53 | 3495 | .126 |
| **Add meal plan** | 0.0845 | 0.0943 | 0.896 | 3413 | .371 |
| **Meal scanned** | 0.143 | 0.104 | 1.38 | 3309 | .168 |
| **Completed nurse check-in** | 0.0963 | 0.0415 | 2.32 | 4028 | .020 |
| **Progress logging** | 0.308 | 0.0423 | 7.29 | 4292 | <.001 |
| **Recipe favourited** | 0.253 | 0.0945 | 2.67 | 3492 | <.001 |
| **Recipe screen viewed** | 0.207 | 0.0413 | 5.01 | 4008 | <.001 |

In these feature-specific models, progress logging showed the strongest association with greater weight loss over time (b = 0.308, SE = 0.042, P < .001), followed by recipe screen viewing (b = 0.207, SE = 0.041, P < .001), goal setting (b = 0.219, SE = 0.042, P < .001), recipe favouriting (b = 0.253, SE = 0.095, P = .008), calorie target setting (b = 0.126, SE = 0.042, P = .003), and nurse check-in completion (b = 0.096, SE = 0.042, P = .020). Dietitian consultation, meal logging, meal plan addition, and meal scanning were not significantly associated with weight-loss trajectory. Features showing evidence of association with weight-loss trajectory and a clinically interpretable direction of effect were then retained for the reduced multivariable model.

## S7: Comparing model fit for models with each quadratic term

To determine whether quadratic app-feature interactions should be included in the final mixed-effects model, feature-specific model comparisons were conducted. For each app feature, two adjusted mixed-effects models were estimated: one including the app feature × linear time interaction only, and one additionally including the app feature × quadratic time interaction. Models were compared using Akaike Information Criterion (AIC) and likelihood-ratio tests (see Table S8). A negative ΔAIC indicated that the model including the quadratic interaction had better fit than the corresponding linear-interaction model.

#### Table S8: Comparing the model fit with mixed-effect models containing different quadratic terms

| **App-feature added as quadratic term** | **AIC for model with linear term only** | **AIC for model with quadratic terms** | **Change in AIC with addition or quadratic term** | **p-value for Likelihood Ratio test** |
| --- | --- | --- | --- | --- |
| Recipe screen viewed | 86538 | 86536 | -2.27 | 0.039 |
| Meal plan added | 86566 | 86566 | -0.0424 | 0.153 |
| Dietitian consult completed | 86565 | 86566 | 0.922 | 0.299 |
| Goal set | 86517 | 86518 | 1 | 0.317 |
| meal_logged | 86564 | 86565 | 1.14 | 0.354 |
| Completed nurse check-in | 86563 | 86565 | 1.52 | 0.488 |
| Meal scanned | 86564 | 86566 | 1.98 | 0.892 |
| Progress logged | 86486 | 86488 | 1.99 | 0.926 |
| Recipe favourited | 86561 | 86563 | 2 | 0.976 |
| Set calorie target | 86547 | 86549 | 2 | 1 |

LRT = Likelihood ratio test

Across the app features examined, recipe screen viewing was the only feature for which adding a quadratic interaction improved model fit. The model including the recipe screen viewed × quadratic time interaction had a lower AIC than the linear-interaction model (AIC = 86,536 vs 86,538; ΔAIC = -2.27), and the likelihood-ratio test indicated a statistically significant improvement in fit (P = .039). In contrast, adding quadratic interactions for the other app features did not meaningfully improve model fit, with ΔAIC values close to zero or positive and non-significant likelihood-ratio tests. Therefore, the final mixed-effects model retained the quadratic time interaction only for recipe screen viewing, while other retained app features were modelled with linear time interactions only. This approach allowed the model to capture the one nonlinear feature-specific trajectory supported by the data while avoiding unnecessary additional interaction terms.

## S8: Collinearity diagnostics for app features mixed-effect model

Table S9 shows the results of collinearity diagnostics for the mixed-effects models.

#### Table S9: Results of collinearity diagnostics for mixed-effects model (all variables are centred)

| **Term** | **VIF** | **VIF 95% CI** | **Adjusted VIF** | **Tolerance** | **Tolerance 95% CI** |
| --- | --- | --- | --- | --- | --- |
| Phase | 6.44 | [6.26, 6.62] | 2.54 | 0.16 | [0.15, 0.16] |
| Phase squared | 5.54 | [5.39, 5.69] | 2.35 | 0.18 | [0.18, 0.19] |
| Calorie target set | 2.59 | [2.53, 2.65] | 1.61 | 0.39 | [0.38, 0.40] |
| Goal set | 3.48 | [3.40, 3.57] | 1.87 | 0.29 | [0.28, 0.29] |
| Nurse check-in completed | 1.39 | [1.36, 1.42] | 1.18 | 0.72 | [0.71, 0.73] |
| Progress added | 2.72 | [2.65, 2.78] | 1.65 | 0.37 | [0.36, 0.38] |
| Recipe favourited | 1.35 | [1.33, 1.38] | 1.16 | 0.74 | [0.72, 0.75] |
| Recipe screen viewed | 3.38 | [3.30, 3.47] | 1.84 | 0.30 | [0.29, 0.30] |
| Drug type | 1.35 | [1.33, 1.38] | 1.16 | 0.74 | [0.73, 0.75] |
| Gender | 1.42 | [1.40, 1.45] | 1.19 | 0.70 | [0.69, 0.72] |
| Age | 1.04 | [1.03, 1.06] | 1.02 | 0.96 | [0.94, 0.97] |
| Ethnicity | 1.06 | [1.04, 1.08] | 1.00 | 0.95 | [0.93, 0.96] |
| Baseline BMI | 1.04 | [1.02, 1.06] | 1.02 | 0.97 | [0.95, 0.98] |
| Phase × Calorie target set | 2.58 | [2.52, 2.64] | 1.61 | 0.39 | [0.38, 0.40] |
| Phase × Goal set | 3.49 | [3.40, 3.58] | 1.87 | 0.29 | [0.28, 0.29] |
| Phase × Nurse check-in completed | 1.39 | [1.37, 1.42] | 1.18 | 0.72 | [0.70, 0.73] |
| Phase × Progress added | 2.74 | [2.67, 2.81] | 1.65 | 0.37 | [0.36, 0.37] |
| Phase × Recipe favourited | 1.36 | [1.33, 1.38] | 1.16 | 0.74 | [0.72, 0.75] |
| Phase × Recipe screen viewed | 8.03 | [7.81, 8.26] | 2.83 | 0.12 | [0.12, 0.13] |
| Phase squared × Recipe screen viewed | 5.37 | [5.23, 5.52] | 2.32 | 0.19 | [0.18, 0.19] |
| Phase × Drug type | 1.76 | [1.72, 1.80] | 1.33 | 0.57 | [0.56, 0.58] |
| Phase × Gender | 1.84 | [1.80, 1.88] | 1.36 | 0.54 | [0.53, 0.55] |

## S9: Remaining diagnostics for app features model

#### Figure S1: Diagnostic models for the mixed-effects model

## S10: Results of full mixed-effects model

Table S10 shows the full-results for the mixed-effects model, including the covariates.

#### Table S10: Full linear mixed-effects model predicting percentage weight loss over time in the App + medication group

| **Predictor** | **Estimate** | **SE** | **df** | **t** | **P value** | **95% Confidence interval** |
| --- | --- | --- | --- | --- | --- | --- |
| **Overall trajectory** |  |  |  |  |  |  |
| Intercept | -0.22 | 0.38 | 5800 | -0.57 | 0.567 | -0.96, 0.52 |
| Time | 3.04 | 0.05 | 12550 | 58.85 | <.001 | 2.94, 3.14 |
| Time² | -0.18 | 0.01 | 10190 | -25.68 | <.001 | -0.20, -0.16 |
| **Baseline differences** |  |  |  |  |  |  |
| Set calorie target (centered) | 0.14 | 0.16 | 4488 | 0.92 | 0.359 | -0.17, 0.45 |
| Set goal (centered) | 0.20 | 0.18 | 4890 | 1.15 | 0.249 | -0.15, 0.55 |
| Nurse check-in completed (centered) | -0.07 | 0.11 | 4796 | -0.67 | 0.502 | -0.29, 0.15 |
| Progress logged (centered) | 0.07 | 0.16 | 5233 | 0.42 | 0.676 | -0.24, 0.38 |
| Recipe favourited (centered) | -0.56 | 0.26 | 4230 | -2.14 | 0.032 | -0.05, -1.07 |
| Recipe viewed (centered) | 0.15 | 0.17 | 8560 | 0.85 | 0.393 | -0.18, 0.48 |
| Initial medication type | 0.64 | 0.12 | 4716 | 5.32 | <.001 | 0.40, 0.88 |
| Gender | 0.63 | 0.12 | 4883 | 5.13 | <.001 | 0.39, 0.87 |
| Age | -0.01 | 0.00 | 5642 | -1.44 | 0.151 | -0.01, -0.01 |
| Baseline BMI | -0.02 | 0.01 | 5507 | -2.10 | 0.035 | -0.04, -0.00 |
| **Ethnicity, compared with Caucasian** |  |  |  |  |  |  |
| Aboriginal or Torres Strait Islander | -0.10 | 0.28 | 5715 | -0.34 | 0.737 | -0.65, 0.45 |
| African | -0.09 | 0.46 | 5633 | -0.19 | 0.848 | -0.99, 0.81 |
| Asian | -0.36 | 0.22 | 5746 | -1.63 | 0.104 | -0.79, 0.07 |
| Latino/Hispanic | -0.21 | 0.38 | 5713 | -0.55 | 0.585 | -0.95, 0.53 |
| Middle Eastern | 0.34 | 0.34 | 5572 | 1.01 | 0.313 | -0.33, 1.01 |
| Pacific Islander or Maori | -0.38 | 0.33 | 5779 | -1.16 | 0.248 | -1.03, 0.27 |
| Not listed | -0.03 | 0.18 | 5681 | -0.18 | 0.860 | -0.38, 0.32 |
| **Interactions with time** |  |  |  |  |  |  |
| Set calorie target (centered) × time | -0.10 | 0.06 | 3734 | -1.74 | 0.082 | -0.22, 0.02 |
| Set goal (centered) × time | 0.04 | 0.07 | 4038 | 0.62 | 0.537 | -0.10, 0.18 |
| Nurse check-in completed (centered) × time | 0.03 | 0.04 | 4001 | 0.66 | 0.508 | -0.05, 0.11 |
| Progress logged (centered) × time | 0.26 | 0.06 | 4336 | 4.46 | <.001 | 0.14, 0.38 |
| Recipe favourited (centered) × time | 0.11 | 0.10 | 3469 | 1.18 | 0.238 | -0.09, 0.31 |
| Recipe viewed (centered) × time | -0.13 | 0.10 | 12320 | -1.27 | 0.204 | -0.33, 0.07 |
| Recipe viewed (centered) × time² | 0.03 | 0.01 | 9988 | 2.10 | 0.036 | 0.01, 0.05 |
| Initial medication type × time | 0.30 | 0.04 | 3914 | 6.73 | <.001 | 0.22, 0.38 |

## S11: Sensitivity analyses: Comparing models with and without dose level

Table S11 shows the results of sensitivity analyses where the app-feature model was conducted but this time adding the final dose level to the model.

#### Table S11: Sensitivity analysis: Results of the app feature model with final dose level included as a covariate

| **Predictor** | **Estimate** | **SE** | **df** | **t** | **P value** | **95% CI Lower** | **95% CI Upper** |
| --- | --- | --- | --- | --- | --- | --- | --- |
| **Overall trajectory** |  |  |  |  |  |  |  |
| Intercept | -0.03 | 0.39 | 5776 | -0.09 | .932 | -0.79 | 0.73 |
| Time | 3.05 | 0.05 | 12510 | 58.92 | <.001 | 2.94 | 3.15 |
| Time² | -0.19 | 0.01 | 10170 | -25.82 | <.001 | -0.20 | -0.17 |
| **Baseline differences** |  |  |  |  |  |  |  |
| Set calorie target | 0.14 | 0.16 | 4480 | 0.91 | .366 | -0.16 | 0.44 |
| Set goal | 0.20 | 0.18 | 4883 | 1.17 | .243 | -0.14 | 0.55 |
| Nurse check-in completed | -0.06 | 0.11 | 4788 | -0.57 | .566 | -0.28 | 0.15 |
| Progress logged | 0.09 | 0.16 | 5232 | 0.58 | .564 | -0.22 | 0.39 |
| Recipe favourited | -0.55 | 0.26 | 4223 | -2.10 | .036 | -1.06 | -0.04 |
| Recipe viewed | 0.14 | 0.17 | 8558 | 0.83 | .406 | -0.20 | 0.48 |
| Tirzepatide vs semaglutide | 0.51 | 0.13 | 5040 | 4.04 | <.001 | 0.26 | 0.76 |
| Male vs female | 0.67 | 0.12 | 4914 | 5.47 | <.001 | 0.43 | 0.92 |
| Age | -0.01 | 0.00 | 5635 | -1.20 | .228 | -0.01 | 0.00 |
| Baseline BMI | -0.02 | 0.01 | 5505 | -1.79 | .073 | -0.03 | 0.00 |
| Dose level | -0.17 | 0.05 | 5660 | -3.35 | <.001 | -0.26 | -0.07 |
| **Ethnicity, compared with Caucasian** |  |  |  |  |  |  |  |
| Aboriginal or Torres Strait Islander | -0.13 | 0.28 | 5712 | -0.46 | .649 | -0.69 | 0.43 |
| African | -0.10 | 0.46 | 5627 | -0.21 | .834 | -1.01 | 0.81 |
| Asian | -0.35 | 0.22 | 5742 | -1.58 | .114 | -0.79 | 0.08 |
| Latino/Hispanic | -0.22 | 0.38 | 5708 | -0.59 | .557 | -0.97 | 0.52 |
| Middle Eastern | 0.34 | 0.34 | 5567 | 1.00 | .317 | -0.33 | 1.00 |
| Pacific Islander or Maori | -0.39 | 0.33 | 5774 | -1.19 | .236 | -1.05 | 0.26 |
| Not listed | -0.03 | 0.18 | 5676 | -0.17 | .865 | -0.38 | 0.32 |
| **Interactions with time** |  |  |  |  |  |  |  |
| Set calorie target × time | -0.10 | 0.06 | 3732 | -1.74 | .082 | -0.21 | 0.01 |
| Set goal × time | 0.04 | 0.07 | 4035 | 0.63 | .530 | -0.09 | 0.17 |
| Nurse check-in completed × time | 0.03 | 0.04 | 3999 | 0.65 | .519 | -0.05 | 0.11 |
| Progress logged × time | 0.26 | 0.06 | 4334 | 4.45 | <.001 | 0.15 | 0.37 |
| Recipe favourited × time | 0.11 | 0.10 | 3467 | 1.17 | .240 | -0.08 | 0.30 |
| Recipe viewed × time | -0.13 | 0.10 | 12320 | -1.29 | .197 | -0.32 | 0.07 |
| Recipe viewed × time² | 0.03 | 0.01 | 9991 | 2.12 | .034 | 0.00 | 0.06 |
| Tirzepatide vs semaglutide × time | 0.30 | 0.04 | 3912 | 6.75 | <.001 | 0.21 | 0.39 |
| Male × time | -0.24 | 0.05 | 4056 | -5.26 | <.001 | -0.33 | -0.15 |

Note: confidence intervals were calculated as Estimate ± 1.96 × SE, so they may differ very slightly from profile or Wald intervals produced directly by confint().

Adding the final dose-level to the quadratic app-feature model improved model fit, as indicated by lower AIC and BIC values. Dose level was significantly associated with percentage weight loss, although the direction of this association should be interpreted cautiously because dose may reflect treatment escalation or clinical response rather than a simple baseline exposure. Importantly, adjustment for dose did not materially alter the app-feature findings: progress tracking remained positively associated with greater weight loss over time, and the quadratic interaction between recipe-screen viewing and time remained statistically significant. These findings suggest that the primary app-feature associations were robust to dose adjustment. Dose effects should be interpreted cautiously as dose was likely modified during treatment in response to clinical progress.

## S12: Comparing inverse probability weighted model to original model using the same dataset

Full results of the IPW sensitivity analyses for the mixed-effects model compared to the original results are available in Table S12. The inverse-probability-weighted sensitivity model produced estimates that were substantively consistent with those from the corresponding unweighted model. Progress logging remained associated with a greater rate of percentage weight loss over time in both the unweighted model (β=0.261, 95% CI 0.146 to 0.375, *P*<.001) and the weighted model (β=0.259, 95% CI 0.143 to 0.376, *P*<.001). The quadratic interaction between recipe viewing and time also remained statistically significant in the unweighted model (β=0.030, 95% CI 0.002 to 0.057, *P*=.036) and the weighted model (β=0.032, 95% CI 0.004 to 0.059, *P*=.025). The estimates for the remaining app-feature interactions, medication type, gender, and the overall trajectory were likewise similar across models. These findings indicate that the principal longitudinal results were not materially altered after weighting for differential follow-up and availability of weight measurements.

#### Table S12: Comparing inverse probability weighted model to original model using the same dataset

| **Predictor** | **Original model** | | | **IPW model** | | |
| --- | --- | --- | --- | --- | --- | --- |
|  | **Estimate** | **95% CI** | ***P*** | **Estimate** | **95% CI** | ***P*** |
| **Overall trajectory** |  |  |  |  |  |  |
| Intercept | −0.22 | −0.972, 0.532 | .567 | −0.228 | −0.983, 0.527 | .554 |
| Time | 3.04 | 2.940, 3.140 | <.001 | 3.050 | 2.950, 3.160 | <.001 |
| Time² | −0.18 | −0.199, −0.171 | <.001 | −0.186 | −0.201, −0.172 | <.001 |
| **Baseline differences** |  |  |  |  |  |  |
| Set calorie target, centred | 0.14 | −0.162, 0.447 | .359 | 0.141 | −0.168, 0.451 | .371 |
| Set goal, centred | 0.20 | −0.142, 0.546 | .249 | 0.208 | −0.141, 0.557 | .243 |
| Nurse check-in completed, centred | −0.08 | −0.293, 0.143 | .502 | −0.071 | −0.292, 0.150 | .531 |
| Progress logged, centred | 0.06 | −0.240, 0.370 | .675 | 0.069 | −0.241, 0.378 | .663 |
| Recipe favourited, centred | −0.56 | −1.080, −0.048 | .032 | −0.562 | −1.090, −0.039 | .035 |
| Recipe viewed, centred | 0.15 | −0.192, 0.487 | .393 | 0.169 | −0.174, 0.512 | .335 |
| Tirzepatide vs semaglutide | 0.64 | 0.403, 0.874 | <.001 | 0.647 | 0.409, 0.884 | <.001 |
| Male vs female | 0.63 | 0.389, 0.871 | <.001 | 0.644 | 0.402, 0.887 | <.001 |
| Age | −0.01 | −0.0159, 0.0025 | .151 | −0.0068 | −0.0160, 0.0025 | .152 |
| Baseline BMI | −0.02 | −0.037, −0.001 | .035 | −0.019 | −0.037, −0.002 | .033 |
| **Ethnicity, relative to Caucasian** |  |  |  |  |  |  |
| Aboriginal or Torres Strait Islander | −0.10 | −0.652, 0.461 | .737 | −0.090 | −0.649, 0.469 | .752 |
| African | −0.09 | −1.000, 0.822 | .848 | −0.107 | −1.020, 0.807 | .818 |
| Asian | −0.36 | −0.799, 0.074 | .104 | −0.365 | −0.802, 0.073 | .102 |
| Latino/Hispanic | −0.21 | −0.955, 0.539 | .585 | −0.207 | −0.957, 0.542 | .588 |
| Middle Eastern | 0.34 | −0.323, 1.010 | .313 | 0.323 | −0.345, 0.990 | .343 |
| Not listed | −0.03 | −0.381, 0.318 | .859 | −0.037 | −0.387, 0.314 | .837 |
| Pacific Islander or Māori | −0.38 | −1.040, 0.268 | .248 | −0.388 | −1.040, 0.267 | .245 |
| **Interactions with time** |  |  |  |  |  |  |
| Set calorie target × time | −0.10 | −0.214, 0.013 | .082 | −0.101 | −0.216, 0.015 | .088 |
| Set goal × time | 0.04 | −0.088, 0.169 | .537 | 0.039 | −0.092, 0.170 | .561 |
| Nurse check-in completed × time | 0.03 | −0.054, 0.109 | .508 | 0.026 | −0.057, 0.108 | .539 |
| Progress logged × time | 0.26 | 0.146, 0.375 | <.001 | 0.259 | 0.143, 0.376 | <.001 |
| Recipe favourited × time | 0.11 | −0.075, 0.303 | .238 | 0.114 | −0.079, 0.306 | .248 |
| Recipe viewed × time | −0.13 | −0.322, 0.069 | .204 | −0.142 | −0.339, 0.055 | .157 |
| Recipe viewed × time² | 0.03 | 0.002, 0.057 | .036 | 0.032 | 0.004, 0.059 | .025 |
| Tirzepatide vs semaglutide × time | 0.30 | 0.214, 0.390 | <.001 | 0.300 | 0.211, 0.389 | <.001 |
| Male vs female × time | −0.24 | −0.330, −0.151 | <.001 | −0.246 | −0.337, −0.156 | <.001 |

**Note.** Estimates are unstandardised regression coefficients. Time refers to study phase, coded from 0 to 6, corresponding to baseline and 30-day intervals through day 180. Time² is the squared phase term. App-feature indicators were mean-centred. The weighted and unweighted models used the same outcome-model specification. Inverse-probability weights accounted for the estimated probabilities of continued follow-up and availability of a weight measurement. Semaglutide, female gender, and Caucasian ethnicity were the reference categories. Confidence intervals are 95% confidence intervals

## S13: Post-hoc power analysis for mixed-effects models

For the linear mixed-effects model, the sensitivity of each coefficient was assessed approximately from its observed standard error using the corresponding normal-theory threshold for 80% power. These calculations were used to describe the precision and detectable effect sizes of the completed analyses and were not interpreted as prospective power calculations or as evidence supporting nonsignificant findings.

#### Table S13: Post-hoc power analysis for mixed-effects model

| **Interaction** | **Estimate** | **t** | **Preliminary assessment** |
| --- | --- | --- | --- |
| Calorie target × time | −0.10 | −1.80 | Low observed-effect power |
| Goal × time | 0.04 | 0.59 | Very low observed-effect power |
| Nurse check-in × time | 0.03 | 0.66 | Very low observed-effect power |
| Progress logged × time | 0.27 | 4.56 | High power |
| Recipe favourited × time | 0.11 | 1.12 | Low power |
| Recipe viewed × time | 0.05 | 0.99 | Low power |
| Recipe viewed × time² | 0.03 | 2.09 | Approximately 55% observed-effect power |
| Drug × time | 0.18 | 4.02 | High power |
| Gender × time | −0.25 | −5.36 | Very high power |

**Note.** Observed power was classified descriptively as very low (<20%), low (20%–49%), moderate (50%–79%), high (80%–89%), or very high (≥90%). These categories are interpretive conventions rather than formal statistical thresholds. Because observed power is derived from the observed effect estimate and its standard error, it should be interpreted alongside the effect estimate and confidence interval and not used as evidence in support of a null finding.

Approximate sensitivity calculations for the mixed-effects model indicated that power differed considerably across interaction terms. The model had high sensitivity for the observed progress logging-by-time, medication-by-time and gender-by-time effects, which had t statistics of 4.56, 4.02 and −5.36, respectively. By contrast, the model had limited sensitivity to the smaller calorie target, goal setting, nurse check-in, recipe favouriting and linear recipe-viewing interactions. The quadratic recipe-viewing-by-time interaction also had limited sensitivity at its observed magnitude, despite reaching statistical significance. Accordingly, nonsignificant feature-by-time interactions should not be interpreted as demonstrating the absence of small longitudinal associations.
